# Thermodynamics and Kinetics of the Cathode–Electrolyte Interface in All-Solid-State Li–S Batteries

**DOI:** 10.1021/jacs.2c07482

**Published:** 2022-09-23

**Authors:** Manas Likhit Holekevi Chandrappa, Ji Qi, Chi Chen, Swastika Banerjee, Shyue Ping Ong

**Affiliations:** † Department of NanoEngineering, University of California San Diego, 9500 Gilman Drive, Mail Code 0448, La Jolla, California 92093-0448, United States; ‡ Materials Science and Engineering Program, University of California San Diego, 9500 Gilman Drive, Mail Code 0448, La Jolla, California 92093-0448, United States; ¶ Department of Chemistry, Indian Institute of Technology Roorkee, Roorkee 247667, India

## Abstract

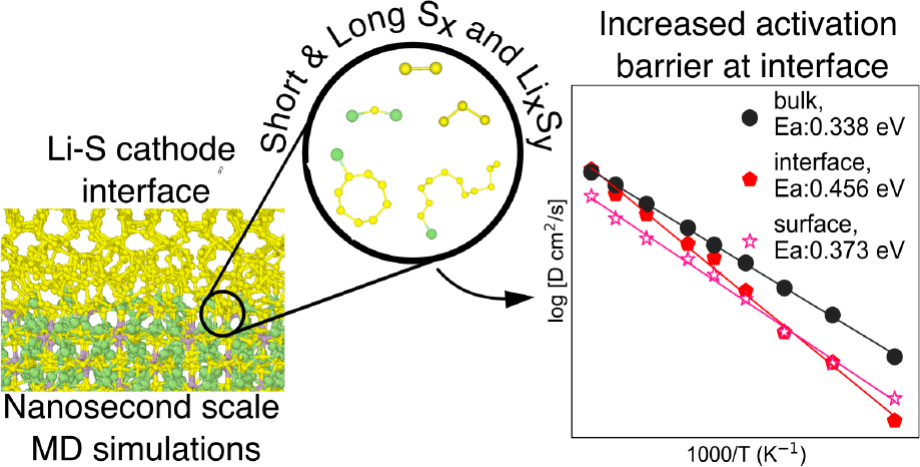

Lithium–sulfur batteries (LSBs) are among the most promising energy storage technologies due to the low cost and high abundance of S. However, the issue of polysulfide shuttling with its corresponding capacity fading is a major impediment to its commercialization. Replacing traditional liquid electrolytes with solid-state electrolytes (SEs) is a potential solution. Here, we present a comprehensive study of the thermodynamics and kinetics of the cathode–electrolyte interface in all-solid-state LSBs using density functional theory based calculations and a machine learning interatomic potential. We find that among the major solid electrolyte chemistries (oxides, sulfides, nitrides, and halides), sulfide SEs are generally predicted to be the most stable against the S_8_ cathode, while the other SE chemistries are predicted to be highly electrochemically unstable. If the use of other SE chemistries is desired for other reasons, several binary and ternary sulfides (e.g., LiAlS_2_, Sc_2_S_3_, Y_2_S_3_) are predicted to be excellent buffer layers. Finally, an accurate moment tensor potential to study the S_8_|β-Li_3_PS_4_ interface was developed using an active learning approach. Molecular dynamics (MD) simulations of large interface models (>1000s atoms) revealed that the most stable Li_3_PS_4_(100) surface tends to form interfaces with S_8_ with 2D channels and lower activation barriers for Li diffusion. These results provide critical new insights into the cathode–electrolyte interface design for next-generation all-solid-state LSBs.

## Introduction

Lithium–sulfur batteries (LSBs) have garnered immense interest as a potential alternative to lithium-ion batteries (LIBs) for future energy storage needs due to their extremely high theoretical capacity (1672 mAh g^–1^)^1,2^ and low cost due to the use of abundant sulfur.^3^ However, commercialization of LSBs still requires solving several challenges that are intrinsic to this technology. While other challenges such as the electrically insulating nature and large volume change during cycling (∼80%) of the S cathode are being addressed via structure and composition engineering,^4−6^ the polysulfide shuttling problem, which is considered to be the most detrimental factor to the cell performance, has not been completely mitigated.

Polysulfide shuttling refers to the concentration gradient-driven shuttling of soluble higher order polysulfides and insoluble lower order polysulfides between the cathode and anode through the liquid electrolyte medium, resulting in irreversible loss of the cathode and anode material. While the formation of polysulfides can be mitigated to some extent with alternative cathode chemistries, these solutions typically result in loss of energy density.^7−9^ Replacing the liquid electrolyte with a solid electrolyte (SE) has been proposed as a potential solution to address the polysulfide shutting problem in LSBs.^10^ For example, gel polymer electrolytes use polar molecules such as polyvinylidene fluoride^11^ (PVDF) and poly(methyl methacrylate)^12^ (PMMA) to trap dissolved polysulfides. In solid polymer electrolytes, the polysulfide migration is inhibited by their adsorption onto inorganic filler surfaces such as Al_2_O_3_^13^ and TiO_2_.^14^ Finally, composite SEs made by dispersing inorganic SEs in poly(ethylene oxide) (PEO)-based electrolytes^15−17^ have been found to have a better ability to inhibit polysulfide movement and improve interfacial stability and mechanical properties when compared to single all-solid polymer or inorganic SEs.^10^ In addition, an all-solid-state LSB architecture may yield other potential advantages of improved system-level energy density and safety.^18,19^

In contrast to all-solid-state LIBs,^20−27^ the interfaces in all-solid-state LSBs, especially that between the SE and the S cathode, have not been extensively studied. The volatility of sulfur makes experimental characterization of these interfaces using traditional tools such as scanning electron microscopy and transmission electron microscopy challenging.^28−30^ Nevertheless, existing studies suggest that these interfaces might not be stable. Zheng et al.^31^ found that the Li_6_PS_5_Cl/S interface undergoes reductive degradation to form Li_3_P, LiP, Li_3_P_7_, or LiP_7_ along with Li_2_S and LiCl. It also undergoes oxidative degradation, resulting in thiosulfate oligomerization, which releases polysulfides that lead to sluggish Li-ion transport and cathode overpotential. An *in situ* study of a PEO-Li_6.75_La_3_Zr_1.75_Ta_0.25_O_12_ polymer ceramic composite interface with sulfur also showed the formation of several polysulfides, which migrate across the SE toward the Li anode.^32^ While *ab initio* molecular dynamics (AIMD) simulations have been successfully used to probe the interfacial chemistries of solid-state (SS) interfaces in LIBs and LSBs,^33,34^ their high computational cost severely restricts the size and the time scale of interfacial simulations. For instance, Camacho-Forero and Balbuena^34^ found that the β-Li_3_PS_4_/cathode interface exhibits oligomerization of PS_4_^3–^ along with formation of various polysulfides within a 50 ps time scale, but interfacial strains of >10% were used to reduce the model size.

In this work, we aim to comprehensively investigate the electrochemical and chemical stability of cathode/SE interfaces in LSBs. We have intentionally not included the Li anode in our study given that this interface, which is no different from that in LIBs, has been extensively studied in prior works.^20−23,33^ Using thermodynamic approximations,^35^ we evaluated a large number of SEs with diverse anion chemistries (from oxides to sulfides to halides) for their interfacial stability against the charged S and discharged Li_2_S cathode. We find that sulfide SEs have the best (electro)chemical stability against the cathode in LSBs among the different classes of SEs. If the use of oxide/halide SEs in LSBs is desired for other reasons (e.g., potential stability against a Li metal anode), we also identified several sulfides that can potentially be used as buffer layers between the cathode and oxide/halide SEs. Finally, we developed a highly accurate machine learning interatomic potential (ML-IAP) via active learning for the β-Li_3_PS_4_|α-S_8_ model interfacial system. Molecular dynamics (MD) simulations on realistic large-scale (thousands of atoms) interfacial simulations using the developed moment tensor potential (MTP) reveal the formation of Li_*x*_S_*y*_ and S_*x*_ species at the interface. The reaction products were in general found to increase the activation energy (*E*_a_) of Li-ion diffusion at the interface, but the type of β-Li_3_PS_4_ surface interfaced was found to be the predominant factor that determines the interfacial Li-migration barrier.

## Methods

### Material Selection

Table 1 presents the list of all the SEs and buffer layer materials studied in this work. Where available, the crystal structures and precomputed energies were obtained from the Materials Project (MP).^36^ Otherwise, the initial crystal structures were obtained from the Inorganic Crystal Structure Database (ICSD).^37^Table S1 provides the sources of SEs considered in this study. We have attempted to be comprehensive in the selection of SE materials, covering all major anion chemistries. For the cathode, we considered both the fully charged (α-S_8_, mp-96) and discharged state (Li_2_S, mp-1153). For buffer layers, we considered all major experimentally reported oxide buffer layers^38^ along with their sulfide analogues with an MP energy above convex hull (*E*_hull_) of <30 meV. Tables S2 and S3 provide the sources and *E*_hull_ values of the oxide and sulfide buffer layers considered in this study.

**Table 1:** List of Solid Electrolytes and Buffer Layers Studied in This Work^a^

category	anion chemistry	compounds
solid electrolytes	oxide	Li_1.3_Al_0.3_Ti_1.7_(PO_4_)_3_,^39^ Li_7_La_3_Zr_2_O_12_ (LLZO), Li_0.33_La_0.56_TiO_3_ (LLTO),^40^ Li_3.5_Zn_0.25_GeO_4_,^41^ Li_2_PO_2_N^42^
	nitride	Li_3_N
	sulfide	Li_4_GeS_4_, Li_10_GeP_2_S_12_ (LGPS), Li_3_PS_4_ (LPS),^43^ Li_7_P_3_S_11_, Li_6_PS_5_Cl (LPSCl)^44^
	halide	Li_3_YCl_6_ (LYC), Li_3_YBr_6_ (LYB)
buffer layers	oxide	Li_4_Ti_5_O_12_, LiNbO_3_ (LNO), LiTaO_3_ (LTO), Li_2_Ti_2_O_5_, Li_4_Ti_5_O_12_, Li_2_ZrO_3_, Li_2_SiO_3_, Li_3_PO_4_, Li_4_TiO_4_, Li_2_TiO_3_, Li_8_Nb_2_O_9_, Li_3_NbO_4_, LiNb_3_O_8_, Li_8_SiO_6_, Li_4_SiO_4_, Li_2_Si_2_O_5_, Li_5_TaO_5_, Li_3_TaO_4_, LiTa_3_O_8_, Li_4_P_2_O_7_, LiAlO_2_, Li_3_BO_3_, LiH_2_PO_4_, LiTi_2_(PO_4_)_3_,LiBa(B_3_O_5_)_3_, LiPO_3_, LiLa(PO_3_)_4_, LiCs(PO_3_)_2_, Al_2_O_3_, ZnO, CdO, Sc_2_O_3_, Y_2_O_3_, La_2_O_3_, SiO_2_, TiO_2_, ZrO_2_, HfO_2_, Nb_2_O_5_, Ta_2_O_5_
	sulfide	Li_2_SiS_3_, Li_3_PS_4_, Li_4_TiS_4_, Li_3_NbS_4_, LiAlS_2_, Li_3_BS_3_, Al_2_S_3_, ZnS, CdS, Sc_2_S_3_, Y_2_S_3_, La_2_S_3_, SiS_2_, TiS_2_, ZrS_2_, HfS_2_

a The corresponding material identifiers are provided in Tables S1, S2 and S3.

For phase diagram construction, the precomputed energies from MP^36^ were used where available. In cases where the material was not part of the MP database (mostly SEs), the structures were obtained from the ICSD database, and DFT computations were carried out. For disordered structures, structure enumeration was performed on supercells to obtain ∼100 ordered candidates, and the lowest energy structure was used for further analysis.

### Thermodynamic Approximations to Interfacial Stability

The electrochemical stability and chemical stability between SEs and LSB cathodes in charged (α-S_8_) and discharged state (Li_2_S) were studied using the well-established fast diffusion of Li-ion and multispecies equilibrium approximations, respectively.^35,45−50^ Here, a brief summary is provided, and interested readers are referred to those previous works for details.

To estimate chemical stability, the reaction energy is calculated by constructing a pseudobinary phase diagram between the SE and cathode and determining the ratio that results in the most negative reaction energy. The assumption here is that the two materials react to form the most favorable products under thermodynamic equilibrium. The reaction energy is given by
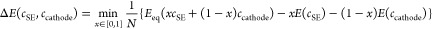
1Here *c*_SE_ and *c*_cathode_ are phases of the SE and cathode, which are the terminal compounds of the pseudobinary phase diagram. *E*(*c*_SE_) and *E*(*c*_cathode_) are their respective density functional theory (DFT) energies. *E*_eq_(*c*) is energy of phase equilibria at a given composition *c*, and *N* is the number of atoms involved in the reaction used as the normalization factor. Δ*E*(*c*_SE_, *c*_cathode_) is the estimate for thermodynamic (equilibrium) stability of the SE and cathode.

The electrochemical stability of the SE/cathode interface was evaluated using the grand potential phase diagram with respect to Li. The interface can be approximated as the SE composition in contact with a Li sink or source. For a system open with respect to Li, the relevant thermodynamic potential is given by the grand potential Φ_eq_, as follows:
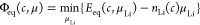
2

The change in the grand potential (ΔΦ) of the SE/cathode interface is given by eq 3 and provides an the estimate of the thermodynamic stability under cycling conditions.
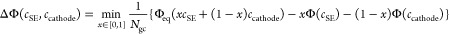
3

It should be noted that the normalization factor *N*_gc_ in eq 3, unlike *N* in eq 1, is the total number of atoms excluding Li. The estimated percentage volume changes (Δ*V*) for thermodynamic reactions were computed using the DFT relaxed volumes of the reactants and products and weighting them in accordance with the ratios in the balanced reaction equation. A positive Δ*V* indicates a net increase in the volume due to the reaction and suggests the possibility of stress buildup and cracking of active material at the interface. A negative Δ*V* indicates a net decrease in the volume due to the reaction, possibly leading to the formation of voids at the interface.

### DFT Calculations

All DFT calculations were performed using the Vienna *Ab initio* Simulation Package (VASP) with the projector augmented wave (PAW) approach.^51,52^ For thermodynamic analysis, the bulk structure relaxations were done using the Perdew–Burke–Ernzerhof (PBE) generalized gradient approximation (GGA) functional,^53^ and the calculation input parameters were kept consistent with MP. The calculations were spin polarized with an energy cutoff of 520 eV and *k*-point density of 64/Å^–3^.

For interface construction, the bulk structures were relaxed with stricter energy and force convergence criteria of 10^–5^ eV and 0.02 eV/Å, respectively. Additionally, for systems containing α-S_8_ (bulk and interface), the relaxation was performed using the optB88 van der Waals (vdW) functional.^54^ The energy cutoff was reduced to 400 eV, and a looser energy convergence criterion of 10^–4^ eV was used.

All the DFT, AIMD simulations, and output analysis were performed using automated workflows^55^ developed using Python Materials Genomics (Pymatgen)^56^ and Fireworks^57^ packages.

### Interfacial Moment Tensor Potential Development

In recent years, machine learning (ML) of the potential energy surface as a function of local environment descriptors^58−65^ has emerged as an automatable approach to develop interatomic potentials (IAPs) for complex chemistries, including SE materials,^66,67^ with near DFT accuracy. In this work, we have adopted the moment tensor potential (MTP) formalism,^61^ which has been shown to provide a good balance between accuracy and computational cost.^68^ Interested readers are referred to those works for details on the MTP formalism.^61^

**Figure 1:**
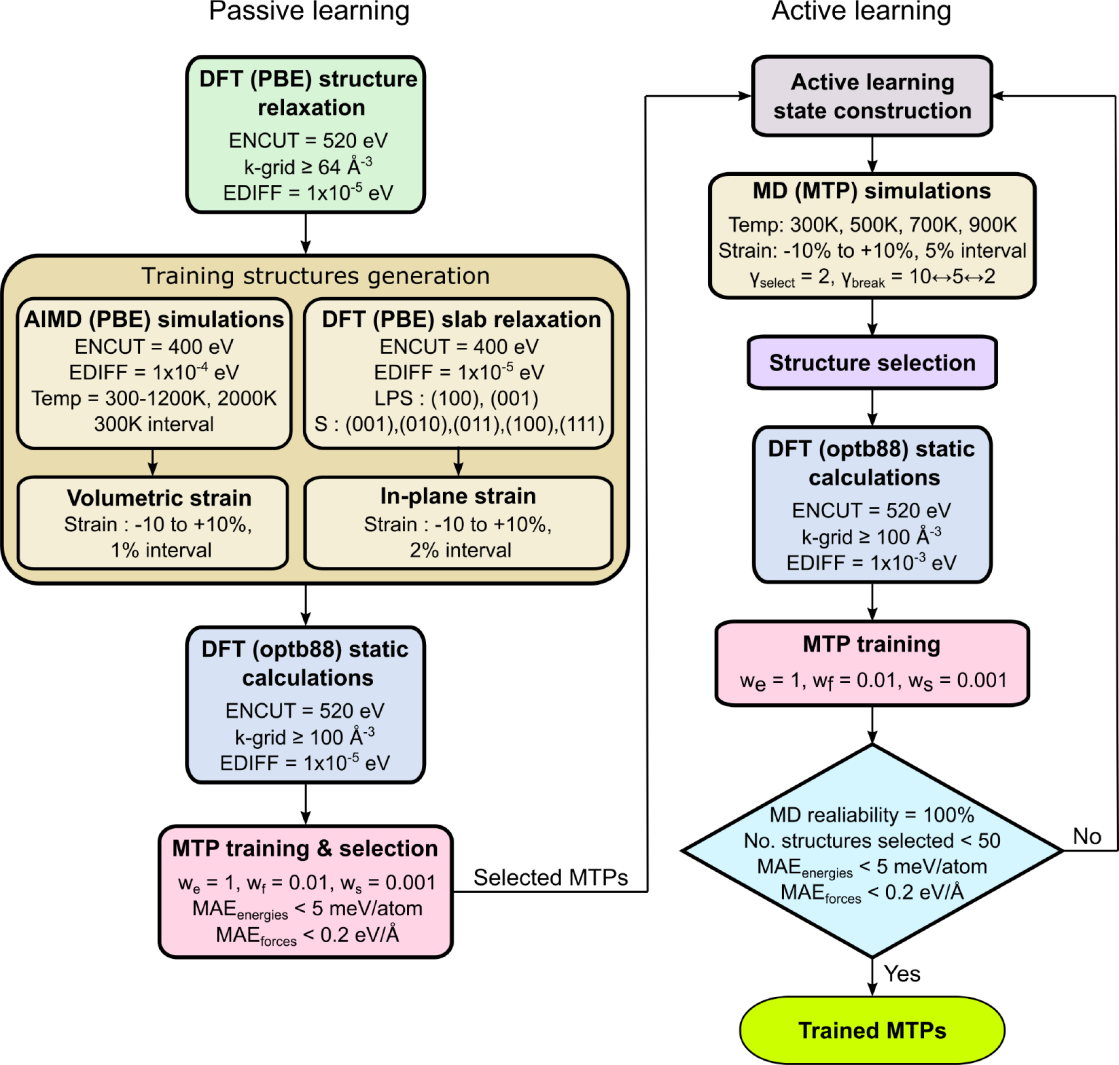
Passive and active learning flowchart for interfacial MTP development.

The α-S_8_|β-Li_3_PS_4_ interface was selected as our model interface of interest. To capture the full complexity of local environments and bonding across the different phases of interest (Li_2_S, α-S, β-Li_3_PS_4_) and at the interface, we adopted a sequential passive learning and active learning workflow adapted from Novikov et al.^69^ to fit the MTP, as shown in Figure 1. In the passive learning stage, training structures were generated by performing non-spin-polarized AIMD simulations using the NVT ensemble on supercells of relaxed bulk structures of β-Li_3_PS_4_, cubic Li_2_S, and α-S_8_ with the PBE functional. Supercells with cell parameters of at least 10 Å were used to minimize the effects of periodic images. A Γ-centered 1 × 1 × 1 *k*-point mesh was used with a plane wave energy cutoff of 400 eV. Temperature control was achieved using a Nose–Hoover thermostat,^70,71^ and the time step was fixed at 2 fs. During the simulation, the 0 K relaxed structures were heated to the target temperature with a temperature gradient of 0.5 K/fs followed by an equilibration period of 10 ps and a production run of 20 ps. The simulations were carried out at 300, 600, 900, 1200, and 2000 K, and 150 snapshots were collected from each temperature at an interval of 0.2 ps, totaling 2250 structures. Further, 10 snapshots were collected at an interval of ∼3 ps at each temperature for Li_2_S and β-Li_3_PS_4_ and volumetrically strained (−10 to +10%, 2% interval) to generate an additional 1000 structures. The training set was further augmented by adding ∼100 slab structures per compound, which were generated by a similar straining procedure of stoichiometric symmetric slabs of these compounds.

Static self-consistent calculations were then performed using the optB88 vdW functional^54^ on the training structures to obtain accurate energies, forces, and stresses. The optB88 vdW functional was selected for the energy and force evaluations given that we expect that vdW forces to play a substantial role in these materials.^66^ Further, the lattice parameters calculated with the optB88 functional for Li_2_S, S, and Li_3_PS_4_ are in much closer agreement with experimental values (see Table S4). The two key parameters that control the trade-off between accuracy and computational cost of MTPs are the radius cutoff (*R*_cutoff_) and the maximum level (lev_max_). MTPs with different radius cutoffs (*R*_cutoff_) and maximum levels (lev_max_) were trained and selected based on convergence of mean absolute error (MAE) for energies (MAE_energies_) and forces (MAE_forces_) of test structures with respect to lev_max_. The training set consisted of 384 training structures, which are tabulated in Table S5. For MTP fitting, a 90:10 training:test split was used with the weights of 100:1:0.1 for energy, force, and stresses. Three MTPs with an *R*_cutoff_ of 5, 6, and 7 Å with a lev_max_ of 20 were chosen for improvement with active learning (AL) (see Figure S1).

For the AL stage, interface models were constructed with slab structures of the different surfaces of β-Li_3_PS_4_ and α-S_8_ using the algorithm proposed by Lazić.^72^Figure S2 shows the slab unit cells used for interface cell construction. The values of DFT surface energies are tabulated in Table S6. For DFT calculations and MTP model training and validation, smaller interfacial models (<250 atoms) were constructed by allowing interfacial mean absolute strains of up to 10% and cell parameters of at least 10 Å along the direction of the interface. Figure S3 shows the seven distinct α-S_8_|β-Li_3_PS_4_ interfaces that satisfy these criteria. Table 2 shows the surfaces used to construct the interfaces, their DFT-computed interfacial energies, and the reference names. The complete details of the parameters associated with the interfaces and their construction are tabulated in Table S7.

**Table 2:** Interfaces Constructed Using Different Surfaces of β-Li_3_PS_4_ and α-S_8_ along with DFT Interfacial Energies

β**-**Li_3_PS_4_ surface	α**-**S_8_ surface	interface energy (J/m^2^)	name
100	001	33.16	S_8_(001)|Li_3_PS_4_(100)
	111	22.88	S_8_(111)|Li_3_PS_4_(100)
001	001	34.15	S_8_(001)|Li_3_PS_4_(001)
	111	41.89	S_8_(111)|Li_3_PS_4_(001)
010	001	39.12	S_8_(001)|Li_3_PS_4_(010)
	001	40.82	S_8_(001)|Li_3_PS_4_(010)(2)
	111	57.58	S_8_(111)|Li_3_PS_4_(010)

A detailed description of the AL process is provided in ref (69). Here, MD simulations were performed at four temperatures (300, 500, 700, and 900 K) on the seven interfacial structures, with three levels of isotropic strain (−5%, 0%, +5%) applied, resulting in 4 × 7 × 3 = 84 MD simulations. The extrapolation grade (γ) is used to select the distinct “new” structures that augment the training set. γ is a measure of the degree of extrapolation the MTP performs to estimate the energy of a structure^73^ and serves as a proxy for energy and force estimation errors.^69^ In practice, two threshold values of γ, γ_select_ and γ_break_, are defined. Structures with γ_select_ < γ < γ_break_ are considered reliable extrapolation and are added to a preselection pool, whereas structures with γ > γ_break_ are deemed to be “risky” and result in termination of simulation.^69^ The γ_select_ and γ_break_ were initially set to 2 and 10, respectively. The γ_break_ value was dynamically changed during iterations between 10 and 2 to ensure no more than 40 000 configurations were selected in any AL iteration. The MD simulations that had no contribution to the preselected structure pool of an iteration were discontinued in future iterations. In addition to reducing computational cost, this tailors the AL workflow toward capturing more diverse and complex local environments, i.e., those generated at higher temperatures and reactive interfaces. Static calculations were performed on the selected structures (a subset of the preselected structure pool) to update the training set and obtain the next iteration of MTP. This process was repeated until the resulting MTP met four conditions: (i) 100% MD reliability (no termination within 1 ns); (ii) fewer than 50 structures were added to the selection pool; (iii) test MAE_energies_ < 5 meV/atom; and (iv) test MAE_forces_ < 0.2 eV/Å. The training and evaluation of MTPs were performed using the MLIP^61^ and Materials Machine Learning^74^ (maml) packages, and MD simulations were performed using the LAMMPS^75^ package.

### Molecular Dynamics Simulations and Analysis

To study the kinetic stability of SE/cathode interfaces, MD NPT simulations were performed using an accurate MTP on large interface models (>1000 atoms) at a 5 ns time scale. An elevated temperature of 600 K was used to speed up the possible chemical reactions at the interface. A 2 fs time step was used, and the interfaces were heated and equilibrated for 40 ps each before the production run of 5 ns. The interface structure evolution and reactions were analyzed using the clustering algorithm implemented in OVITO visualization software.^76^ The molecular nature of α-S_8_ and the interface reaction products allows for the use of a bond connectivity based clustering algorithm to decompose the interfacial structure into molecular clusters.

A cluster is defined as a set of connected points within which every atom is within the reach of every other atom through at least one continuous path. The path is made of atoms and their bonds with the adjacent atoms. Element pairwise bond length cutoff criteria were used to consider a pair of atoms bonded. The cutoffs were set at values 10% higher than the equilibrium bond lengths to account for bond expansion due to heating of the structure in MD simulations. Figure S4 provides examples of clustering of atoms based on the bond length cutoff criteria.

For Li diffusion analysis, the diffusivity (D*) was calculated by performing linear fitting of mean square displacement (MSD) of a Li ion with time using the Einstein relation^77^
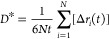
4Here, *N* is the total number of Li ions and Δ*r*_*i*_(*t*) is displacement of the *i*th ion at time *t*. For calculation of surface/interfacial diffusivities, only Li atoms with both start and end positions of hopping inside the surface/interfacial region were considered for MSD calculation.^78−81^ The Arrhenius plot was then obtained to calculate the bulk/surface/interface activation barrier, *E*_a_.

## Results and Discussion

### Thermodynamic Analysis of SE/Buffer/Cathode Interfaces in LSBs

#### Chemical Stability of SE/Cathode Interfaces

**Figure 2:**
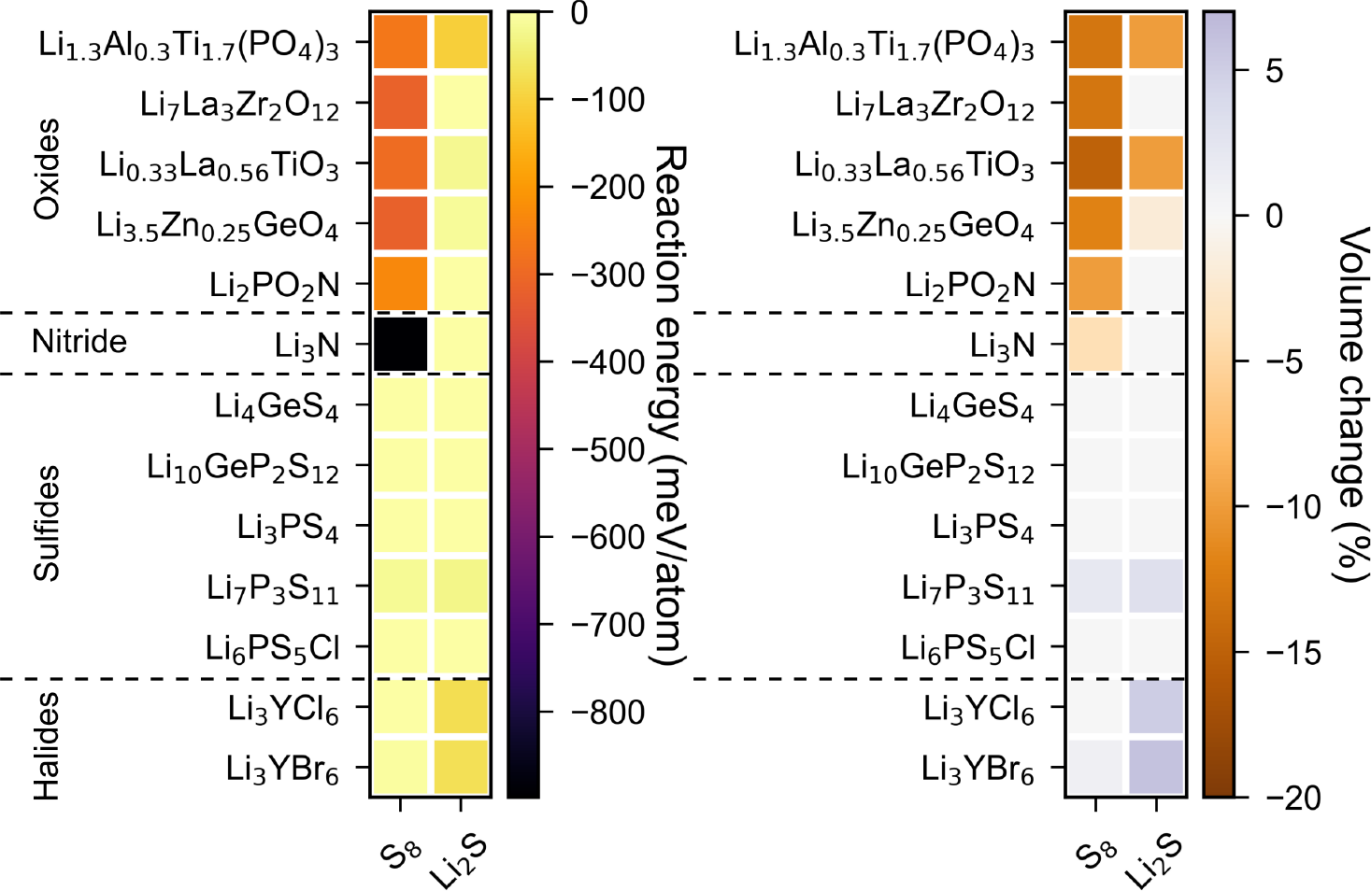
Reaction energy (left) and percentage volume change (right) due to reaction for different electrolyte/electrode pairs.

Figure 2 shows the reaction energy (left) and corresponding volume change (right) for the different SE/electrode under consideration. The complete list of reaction products of all SE/cathode pairs is provided in Table S8. Li_3_N/S_8_ is found to be the most unstable SE/electrode pair with a very high reaction energy of −900 meV/atom. This results in the formation of Li_2_S and the evolution of N_2_, which might present a safety hazard. All oxide SEs are found to be unstable against S_8_ with similar reaction energies and a volume shrinkage of ∼250–300 meV/atom and ∼8–10%, respectively. The reaction products generally include metal sulfides such as LaS_2_, ZrS_3_ (TiS_3_), and Li_2_S. Among the halide SEs, the LYC/S_8_ interface is predicted to be chemically stable. LYB reacts with a small reaction energy of 22 meV/atom and a negligible volume increase of ∼1% to form binary compounds such as Y_2_S_3_, LiBr, and SBr.

The sulfide SE/S_8_ interfaces show the best chemical compatibility except in the case of Li_7_P_3_S_11_, which reacts to form Li_3_PS_4_ and P_2_S_7_. The discharged Li_2_S cathode is generally more chemically stable against all oxide and sulfide SEs compared to the charged S_8_ cathode. Surprisingly, the discharged Li_2_S cathode is chemically less stable against halide SEs when compared to the charged S_8_ cathode. This is due to the formation of highly stable compounds such as LiX (X = Cl, Br) and LiYS_2_.

#### Electrochemical Stability of SE/Cathode Interfaces

**Figure 3:**
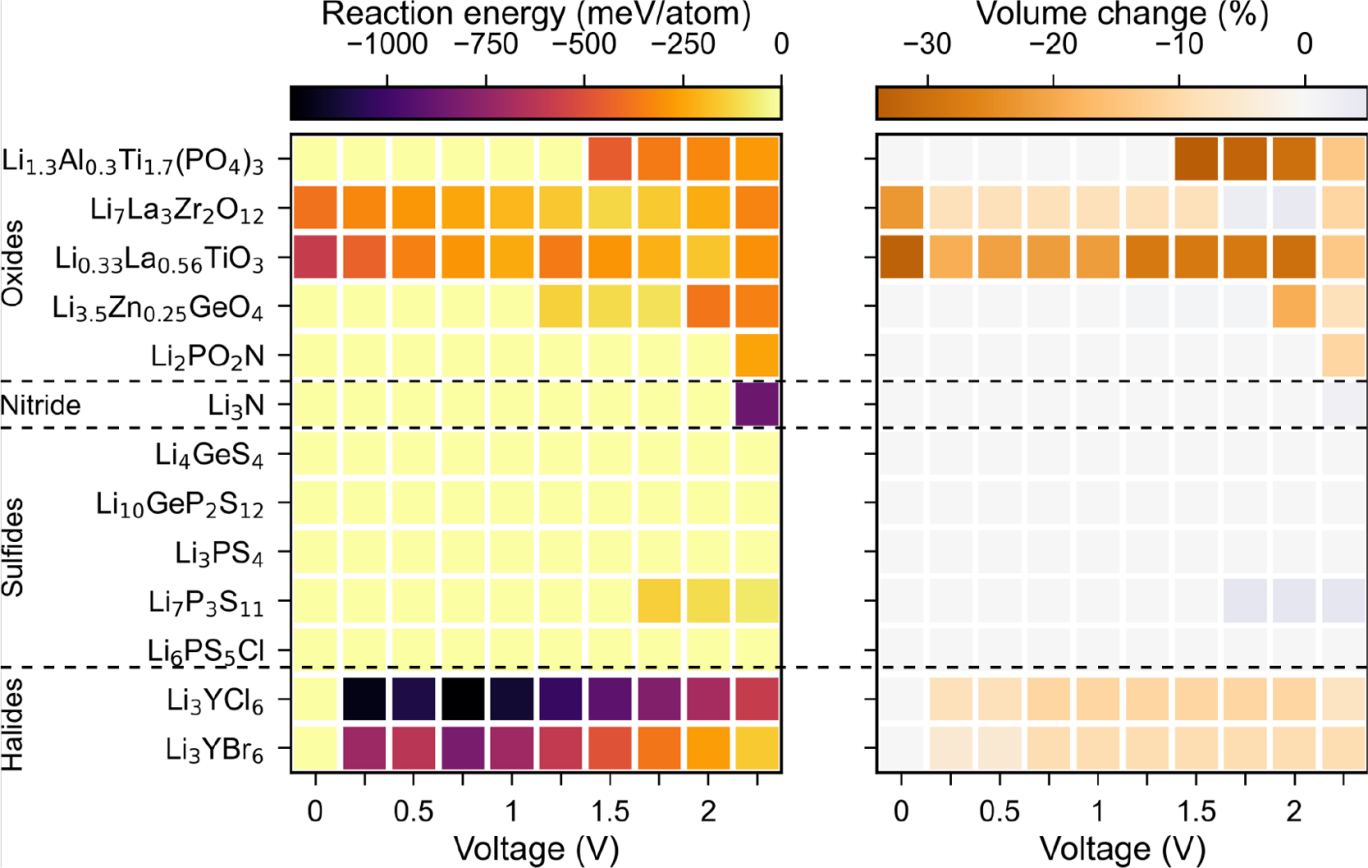
Electrochemical reaction energy (left) and corresponding volume change (right) for α-S_8_.

Figure 3 shows the electrochemical reaction energy (left) and corresponding volume change (right) for SE/α-S_8_ interfaces between 0 and 2.25 V. In general, the qualitative observations about the relative chemical stabilities of different SE anion chemistries also apply to the relative electrochemical stabilities. The Li_3_N/S_8_ interface is electrochemically stable up to 2 V, after which the interface is predicted to decompose to Li and S_7_N with a high reaction energy of −865 meV/atom and volume change of about 2%. The halide SEs interfaces show very limited electrochemical stability and start to react at 0.25 V to form LiX (where X = Cl, Br) and YS with a high reaction energy of >500 meV/atom. However, the volume changes are <10%. Similarly, the oxide LLZO and LLTO interfaces are electrochemically unstable at all voltages. Li_1.3_Al_0.3_Ti_1.7_(PO_4_)_3_ and Li_3.5_Zn_0.25_GeO_4_ have slightly larger stability windows of 0–1.25 V and 0–1 V, respectively. Finally, the sulfide SE interfaces are electrochemically stable at all voltages with the exception of Li_7_P_3_S_11_ at 1.5 V, where it reacts with S_8_ to form Li_3_PS_4_.

Compared to the charged S_8_ cathode, the discharged Li_2_S cathode/SE interfaces have identical reactions with SEs, but the reaction energies and resultant volume changes are in general predicted to be significantly lower than α-S_8_, as shown in Figure S5.

#### Chemical Stability of SE/Buffer Layer Interfaces

**Figure 4:**
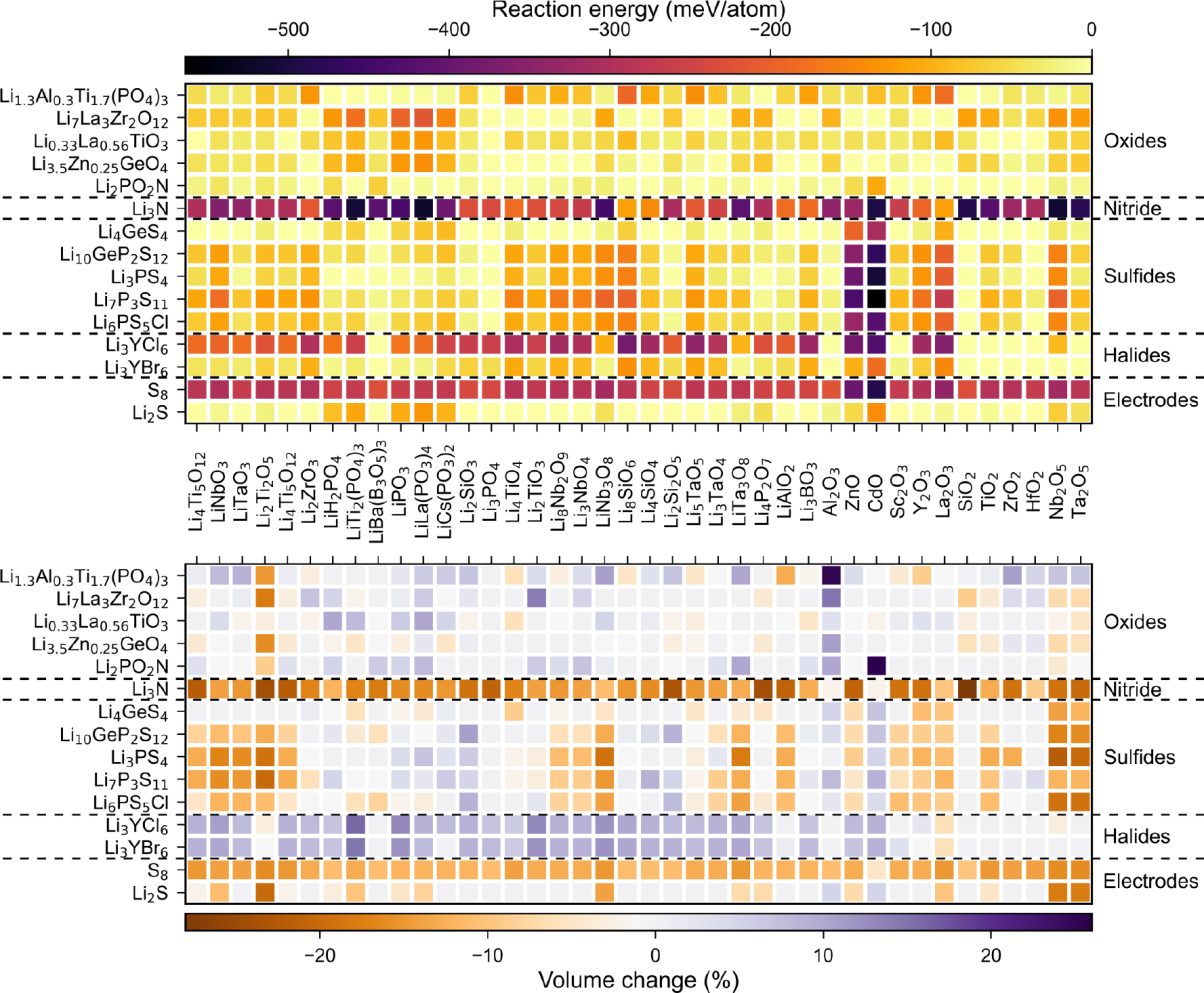
Reaction energy (top) and volume change (bottom) for different electrolyte and electrode with oxide buffer layer material pairs.

**Figure 5:**
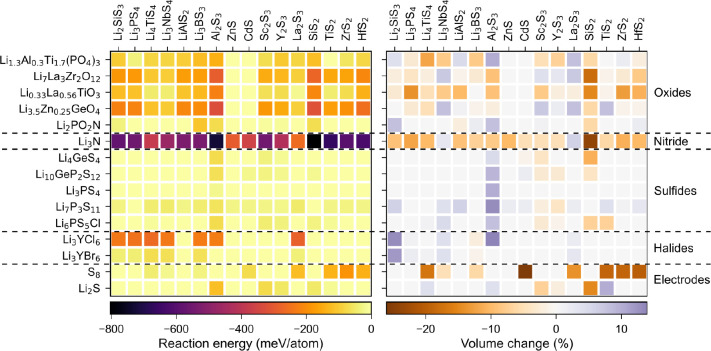
Reaction energy (left) and volume change (right) for different electrolytes and electrodes with sulfide buffer layer material.

In general, oxide and halide SEs are predicted to be both chemically and electrochemically unstable against LSB cathodes to varying degrees. Nevertheless, there may be potential situations where the use of an oxide or halide SE may be desired. For instance, oxide SEs may potentially provide better chemical and mechanical compatibility with the Li metal anode. Here, we investigate potential oxide or sulfide buffer layers that can be used to stabilize interfaces. Figures 4 and 5 show the predicted reaction energies and corresponding percentage volume changes for SEs and cathode interfaces with oxide and sulfide buffer materials.

As can been seen, like oxide SEs, all the oxide buffer materials are predicted to be incompatible with a S_8_ cathode. However, some sulfides show promise of being used as buffer layers. For example, some binary and ternary sulfides such as Sc_2_S_3_, Y_2_S_3_, Li_3_PS_4_, and LiAlS_2_ are predicted to have chemical compatibility with both Li_2_PO_2_N and the charged and discharged LSB cathodes. The binary sulfides also exhibit good chemical compatibility with halide SEs. Li_3_PS_4_ can also be used to buffer the Li_1.3_Al_0.3_Ti_1.7_(PO_4_)_3_ interfaces as well.

### Atomistic Studies of the S_8_/β-Li_3_PS_4_ Interface

Given that sulfide SEs appear to be the most stable against the S_8_ cathode in LSBs, we have selected the β-Li_3_PS_4_/S_8_ interface as our model system for in-depth studies of interfacial kinetics via MD simulations using a fitted MTP. In the following sections, we will first provide a validation of our fitted MTP before analyzing the results of the MD simulations.

#### MTP Validation

**Figure 6:**
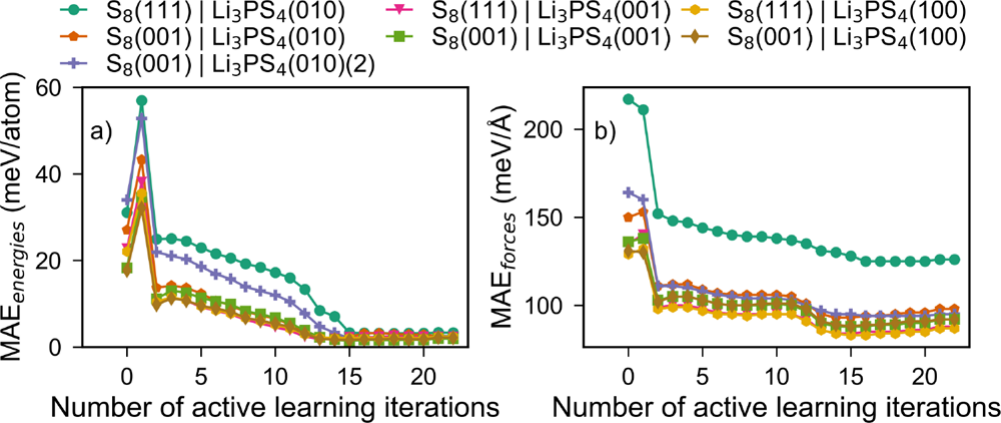
(a) MAE_energies_ and (b) MAE_forces_ for test structures obtained from different interfaces during active learning iterations. The test structures were collected from MD simulations performed at 300, 500, 700, and 900 K of the last iteration AL to ensure no structures were part of the training set (added back during AL).

Figure 6a and b show the evolution of the test MAE_energies_ and MAE_forces_ for different interfaces during the AL process. The test structures were generated by production MD simulations at 300 and 600 K using the MTP trained at the last (25th) iteration of AL. Test structures were collected at 2 ps intervals during the heating and equilibration stages and at 20 ps intervals during the production stage. It may be observed that the test MAE_energies_ and MAE_forces_ converge to below 5 meV atom^–1^ and 150 meV Å^–1^, respectively, for all interfaces after about 15 AL iterations. The fitted MTP is also able to reproduce the lattice parameters and relative energy differences for the α, β, and γ polymorphs of Li_3_PS_4_, as shown in Table S9.

**Figure 7:**
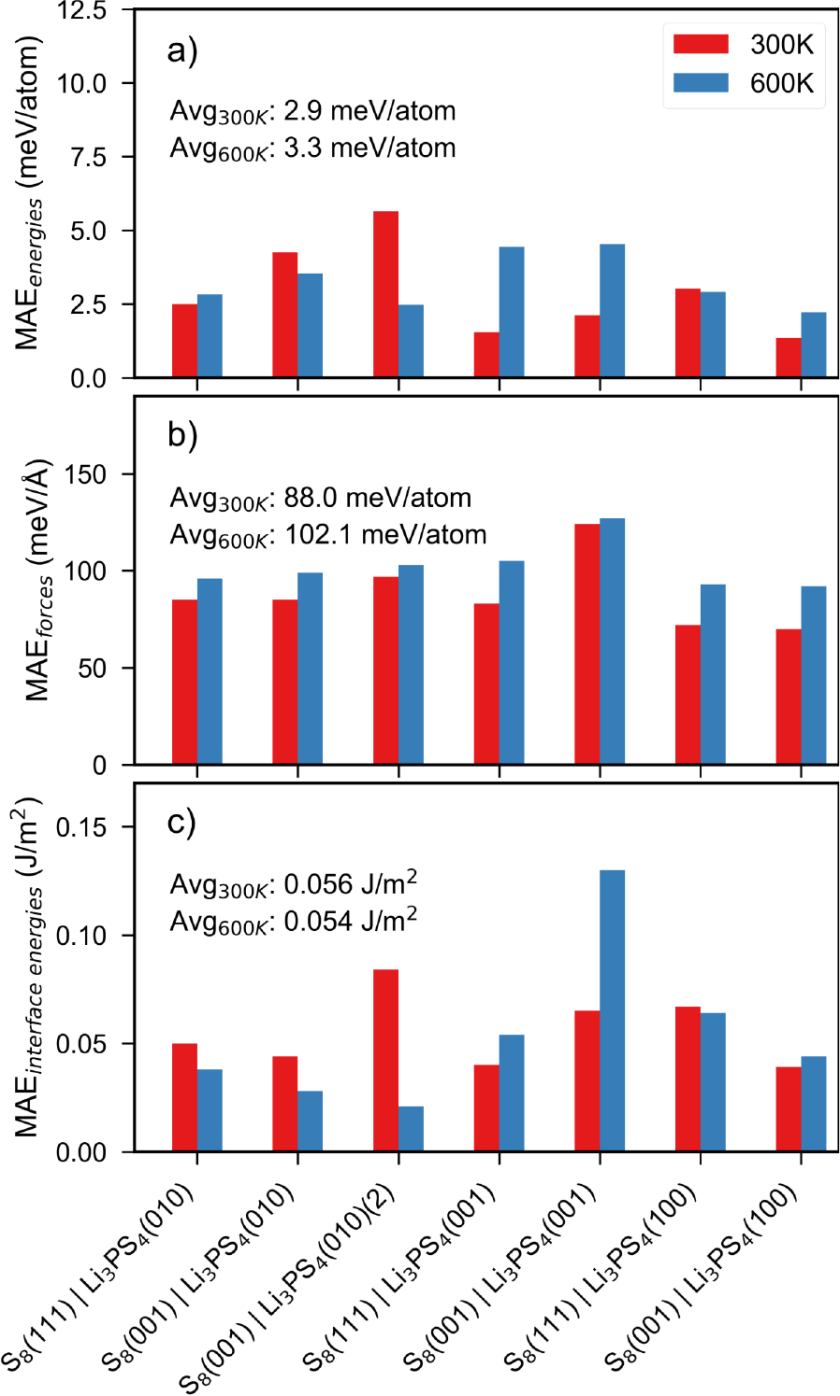
MAE of MTP-predicted (a) energies, (b) forces, and (c) interfacial energies relative to DFT values for test structures collected from 300 and 600 K production run MD simulations.

Figure 7a–c compare the test MAE_energies_ and test MAE_forces_ as well as calculated MAE_interface energies_ for all the interfaces. Across all interfaces, the MAE_energies_ and MAE_forces_ are consistent with the results obtained during the AL iterations. The MAE_interface energies_ are within 0.15 J m^–2^. It is important to note that the MTP was not trained on MD structures from 600 K during AL, and yet the errors for 600 K structures are low. This indicates that the MTP is unlikely to be overfitted.

Table 3 compares the DFT and MTP-computed cell constants, mechanical properties, and surface energies for β-Li_3_PS_4_ and α-S_8_. In general, we observe that the errors in the cell constants and mechanical properties are extremely low. However, the MTP tends to underestimate surface energies for both β-Li_3_PS_4_ and α-S_8_. The MTP-predicted surface energies for α-S_8_ are well within 0.05 J/m^2^, possibly due to their lower bonding complexity as well as the lower absolute values of the surface energies. The errors in the MTP-predicted surface energies of the more complex and higher-energy β-Li_3_PS_4_ surfaces are somewhat higher, but in line with the MAE in interface energies during the AL iterations.

**Table 3:** Comparison of DFT- and MTP-Computed Cell Parameters, Mechanical Properties, and Surface Energies of β-Li_3_PS_4_ and α-S_8_^a^

	β-Li_3_PS_4_		α-S_8_	
quantity	DFT	MTP	DFT	MTP
Cell Parameters				
*a* (Å)	13.07	12.99 (−0.08)	10.33	10.31 (−0.02)
*b* (Å)	8.13	8.09 (−0.05)	12.83	12.80 (−0.03)
*c* (Å)	6.26	6.23 (−0.03)	24.5	24.44 (−0.06)
Mechanical Properties				
bulk modulus (GPa)	22	29.61 (7.61)	14.34	20.25 (5.91)
shear modulus (GPa)	11	14.81 (3.81)	7.28	11.77 (4.48)
Poisson’s ratio (GPa)	0.27	0.32 (0.05)	0.28	0.28 (0.00)
Surface Energies (J/m^**2**^)				
surface index				
100	0.371	0.321 (−0.050)	0.172	0.151 (−0.021)
001	0.608	0.449 (−0.159)	0.188	0.157 (−0.031)
010	0.620	0.560 (−0.060)	0.185	0.172 (−0.013)
111			0.153	0.132 (−0.021)

a Values in parentheses show the absolute error of MTP-computed values relative to the DFT values.

#### MD Simulations of β-Li_3_PS_4_/S_8_ Interfaces

**Figure 8:**
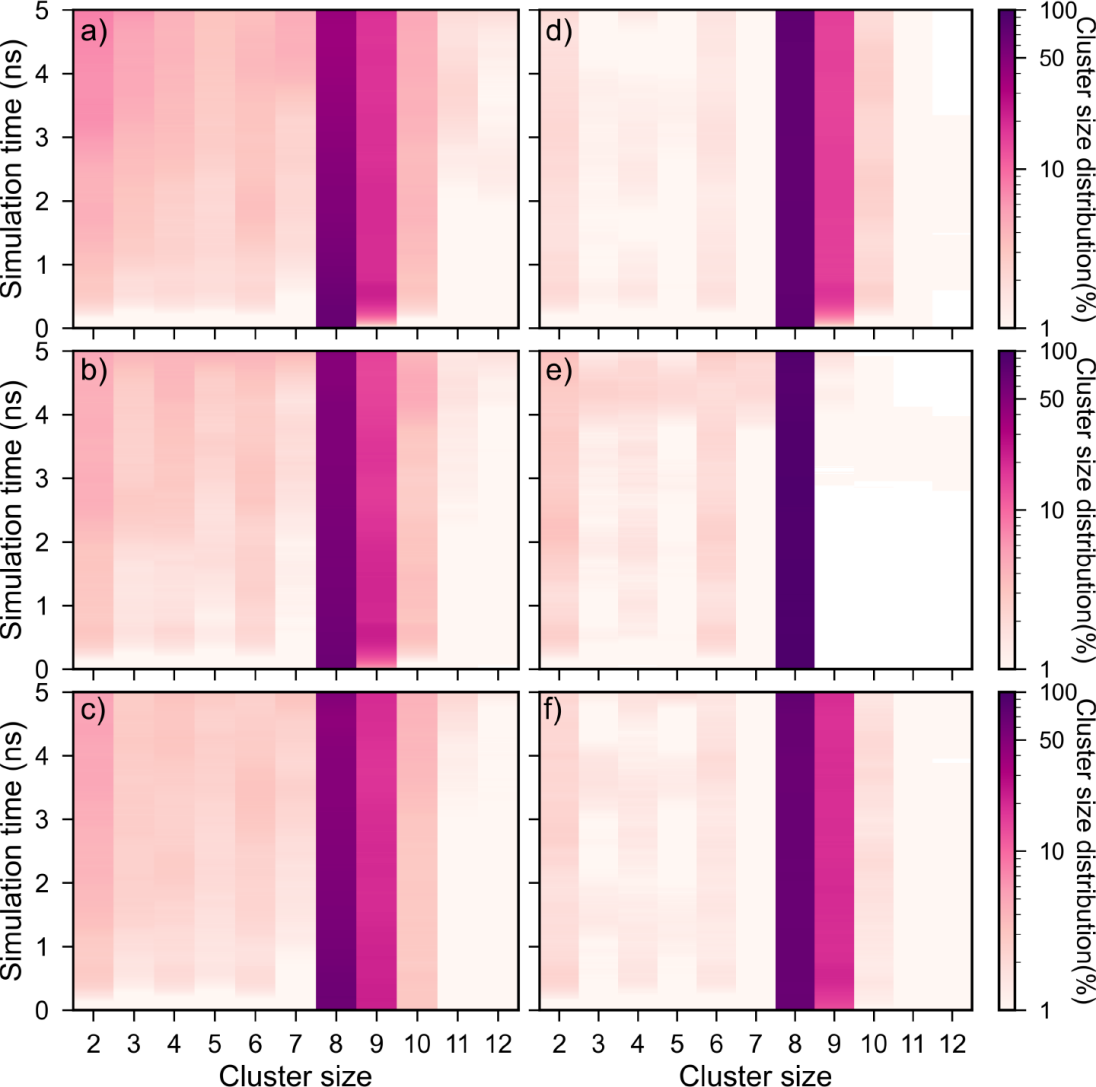
Time evolution of cluster size distribution in (a) S_8_(001)|Li_3_PS_4_(001), (b) S_8_(001)|Li_3_PS_4_(010), (c) S_8_(001)|Li_3_PS_4_(100), (d) S_8_(111)|Li_3_PS_4_(001), (e) S_8_(111)|Li_3_PS_4_(010), and (f) S_8_(111)|Li_3_PS_4_(100) interfaces.

**Figure 9:**
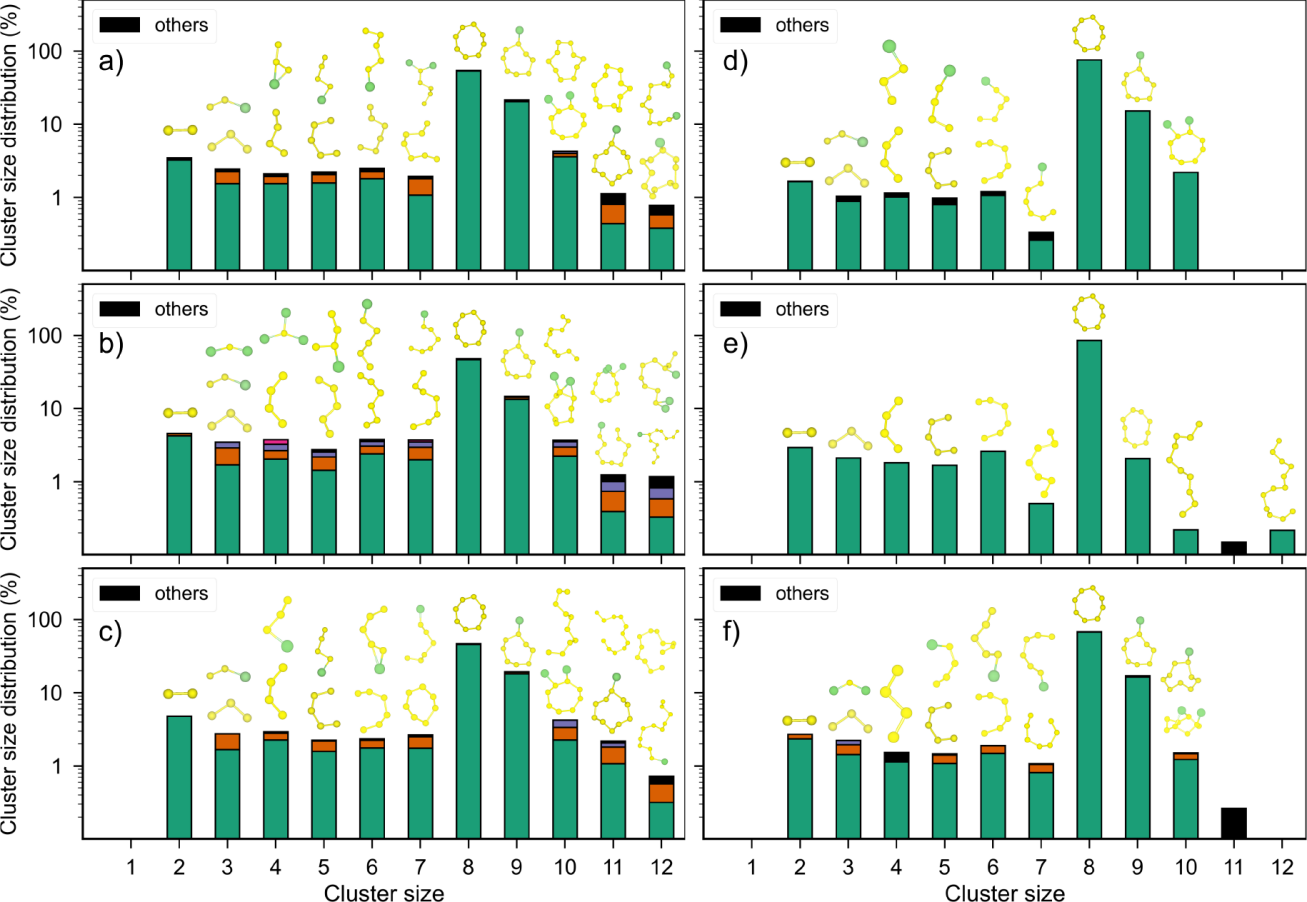
Averaged cluster size distribution and composition at (a) S_8_(001)|Li_3_PS_4_(001), (b) S_8_(001)|Li_3_PS_4_(010), (c) S_8_(001)|Li_3_PS_4_(100), (d) S_8_(111)|Li_3_PS_4_(001), (e) S_8_(111)|Li_3_PS_4_(010), and (f) S_8_(111)|Li_3_PS_4_(100) interfaces.

Figure 8a–f show the time evolution of cluster size (*n*_c_) distribution of β-Li_3_PS_4_ interfaces with the S_8_ surfaces with the highest (left panels) and lowest (right panels) surface energies, i.e., the (001) and (111) surfaces, respectively. It should be noted that no phase transformation from β-Li_3_PS_4_ to either γ-Li_3_PS_4_ or α-Li_3_PS_4_ was observed within the MD simulation time scale, as such transformations require a kinetically activated rearrangement of PS_4_ and LiS_4_/LiS_6_ polyhedra. In general, it can be seen that the percentage of clusters with *n*_c_ < 8 increases steadily over the simulation time with a concurrent decrease in the percentage of clusters with *n*_c_ = 8 (S_8_ molecules), indicating the breakdown of S_8_ rings into smaller S rings/chains. However, there is a noticeable difference in the evolution of α-S_8_ (001) and (111) based interfaces. The clusters with *n*_c_ ≠ 8 form sooner and at much higher concentrations at the S(001)-based interfaces (Figure 8a–c). There is also a significantly smaller percentage of clusters with *n*_c_ > 9 at the S(111)-based interfaces (Figure 8d–f). Figure 9a–f show the average cluster size *n̅*_c_ distribution and composition within each cluster size for the β-Li_3_PS_4_ interfaces with the S_8_ surfaces with the highest and lowest surface energies, i.e., the (001) and (111) surfaces, respectively. The averaging was done over the last 1 ns of simulation. The molecular structures constituting a given cluster size are shown on top of each cluster size in the same order as their percentage subcomposition shown in cluster size bars. For example, in Figure 9a, the *n*_c_ = 3 clusters consist of S_3_ and LiS_2_ type molecules at ∼1% and ∼0.1% composition, respectively. The clusters that were present at less than 0.1% concentration are classified as “others”. It is also important to note that Li atoms remain bound to β-Li_3_PS_4_ and any molecular cluster containing Li should be thought of as S rings/chain/atom interacting with the surface Li atoms of β-Li_3_PS_4_ and not as free-floating Li-polysulfides.

While there are some differences in the percentage composition of the different interfaces, the qualitative trends in cluster distribution profiles are remarkably similar. Each cluster size predominantly consists of S rings/chains at 2–3% followed by the presence of a small percentage (<1%) of Li polysulfide-like (Li–S_*x*_) molecules. The Li–S_*x*_ molecules are formed by the interaction of S_*x*_ rings/chains with the Li ions from β-Li_3_PS_4_ present at the interfaces.

Based on the bond lengths and bond angles (accounting for thermal expansion of bonds), the *n*_c_ = 3 clusters strongly resemble the known structures of S_3_ (S_3_ trimer) and solid-state c-Li_2_S. The *n*_c_ = 2 S_2_ clusters also closely resemble the known S_2_ dimer structure. While the presence of shorter Li–S_*x*_ (*x* < 8) molecules can be expected at the interface, the presence of larger Li–S_*x*_ (*x* > 8) molecules at a similar percentage composition is interesting. These larger Li–S_*x*_ (*x* > 8) molecules are formed by the fusion of smaller S_*x*_ chains followed by absorption onto the β-Li_3_PS_4_ surface at the interface. This might indicate a fundamental difference in the interface composition between liquid and solid-state Li–S batteries.

From Figure 9d–f, it may be observed that the percentage of smaller clusters (*n*_c_ < 8) is much lower (<1%) for the S_8_(111) interfaces compared to the S_8_(001) interfaces; that is, the S_8_(111) interfaces are less reactive than the S_8_(001) interfaces. This is likely due to the fact that the (111) surface is significantly lower in energy and, hence, more stable than the (001) surface. This is also supported by the plateauing of the percentage of cluster sizes at ∼2% in Figure S7a–f, which is not observed in the case of the (001) surface (Figure S6a–f). As found in the time evolution analysis, there is a marked decrease in the presence of larger Li–S_*x*_ (*x* > 10) polysulfide resembling structures. However, the types of shorter S rings/chains and Li–S_*x*_ (*x* < 8) polysulfide structures are similar to that of α-S_8_(001) interfaces. It is interesting to note the similarities and differences in the interfacial reaction products from a previous AIMD study on the same kind of interface.^34^ While the formation of a few smaller S_*x*_ species such as S dimers and trimers was shown, this study found no evidence of the formation of larger S_*x*_ and Li_*x*_S_*y*_ types of species. This difference, among other factors, such as interface morphology and interfacial strain, might be due to the limited system size and time scale of the simulations possible using *ab initio* methods. The larger system size and time scale of MTP-driven MD simulations allow for extended periods of chemical interactions of various species at the interface, thus allowing for the formation of larger polysulfide species. This also eliminates the need for using less realistic interfaces with high interfacial strain (used to limit system size), which also presumably impacts the interfacial reactivity.

**Figure 10:**
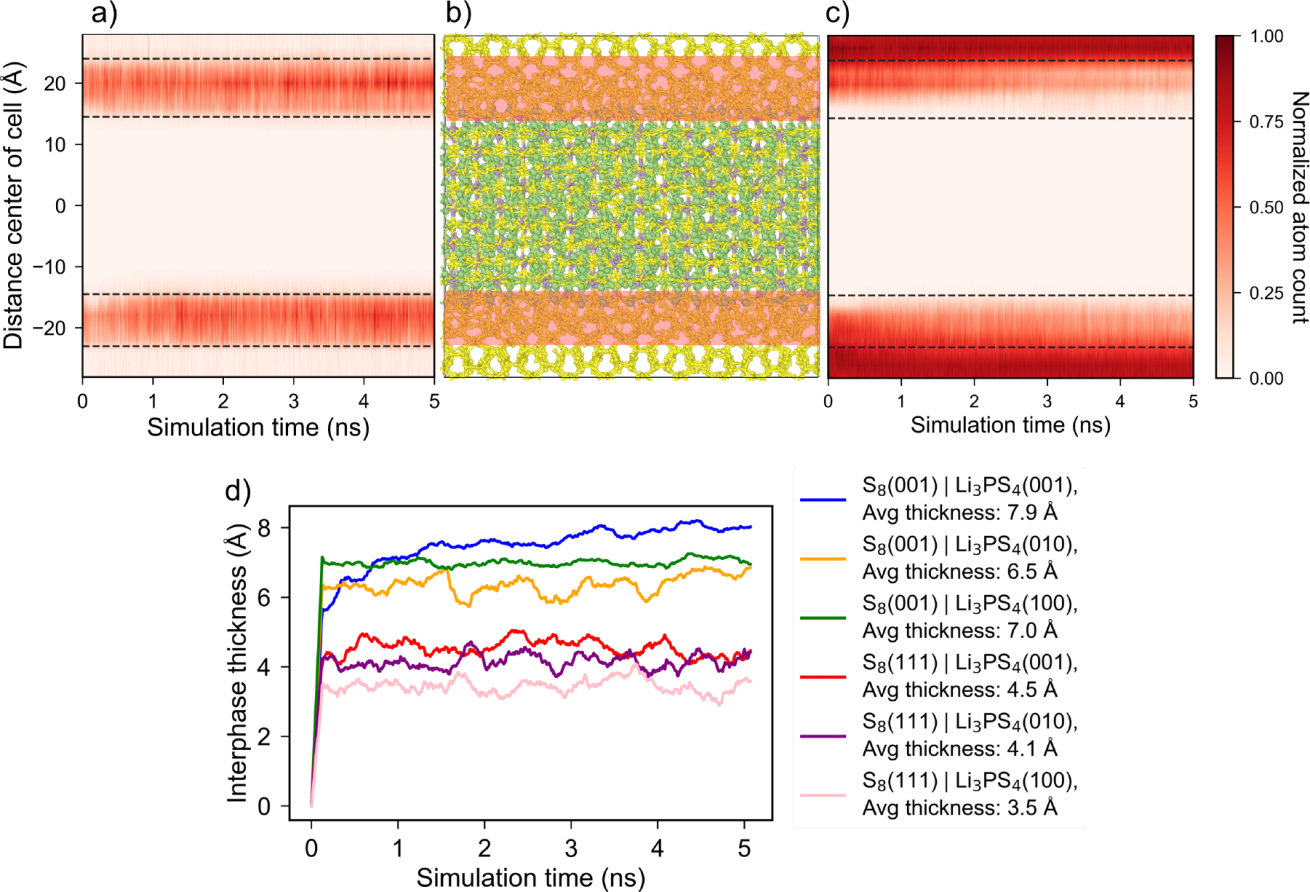
(a) Time evolution of normalized atom count of interface reaction products (cluster size ≠ 8) as a function of the distance for the S_8_(001)|Li_3_PS_4_(001) interface. The center of the bulk Li_3_PS_4_ region is the zero reference. (b) S_8_(001)|Li_3_PS_4_(001) interface cell with highlighted interface region. The Li, P, and S atoms are represented by green, purple, and yellow atoms, respectively. (c) Time evolution of normalized atom count of S_8_ atoms as a function of the distance from the S_8_(001)|Li_3_PS_4_(001) interface. (d) Time evolution of the interphase layer thickness in α-S_8_|β-Li_3_PS_4_ interfaces. The average interphase thickness is averaged over the last 2 ns.

Figure 10a and c show the time evolution of the spatial distribution of non-S_8_ and S_8_ clusters, respectively, as a function of distance along the direction perpendicular to the interface for the S_8_(001)|Li_3_PS_4_(001) interface. The interface structure is shown in Figure 10b. It can be seen that the thickness of the reaction zone (interphase) increases initially, after which it plateaus. There is a concurrent decrease in the concentration of S_8_ ring atoms in the reaction zone. The bulk-like regions of α-S_8_ are unaffected. It is important to note that while the interphase region grows, there is no evidence of diffusion of reaction products (Li polysulfides) into the Li_3_PS_4_ bulk region within the simulated time scale. This is true for all the interfaces under consideration, as evident from Figures S8a–f. Thus, crystalline β-Li_3_PS_4_ is effective in blocking polysulfide diffusion. It should be noted that, unlike a full cell battery, there is no applied electrochemical driving force for the Li from β-Li_3_PS_4_ to diffuse into α-S_8_ except for the difference in the Li chemical potential between the compounds. Further, the presence of grain boundaries at the interface will also impact the interfacial reactivity, interphase thickness, and polysulfide mobility, which will be the focus of a future study.

Figure 10d shows the evolution of interphase thickness for the interfaces during the simulation time scale. The interphase thickness is averaged over the two interfaces present in the interface cell. The time-averaged interphase thickness is calculated over the last 2 ns of the simulation. It can be seen that the interphase grows very rapidly within the first 100 ps for all the interfaces, after which there are only minor fluctuations. The α-S_8_(001) surface based interfaces with higher reactivity form a considerably thicker interphase layer of 6.5–8 Å. The more stable α-S_8_(111)-based interfaces have an interphase thickness of 3.5–4.5 Å. Hence, surface stability can have a profound effect on the interfacial reactivity and, correspondingly, the thickness of the interphase layer. These interphase thicknesses can be considered as the spatial limits of interface-related phenomenon and are used as the “interface region” for the analysis of ionic diffusivities in this region.

**Figure 11:**
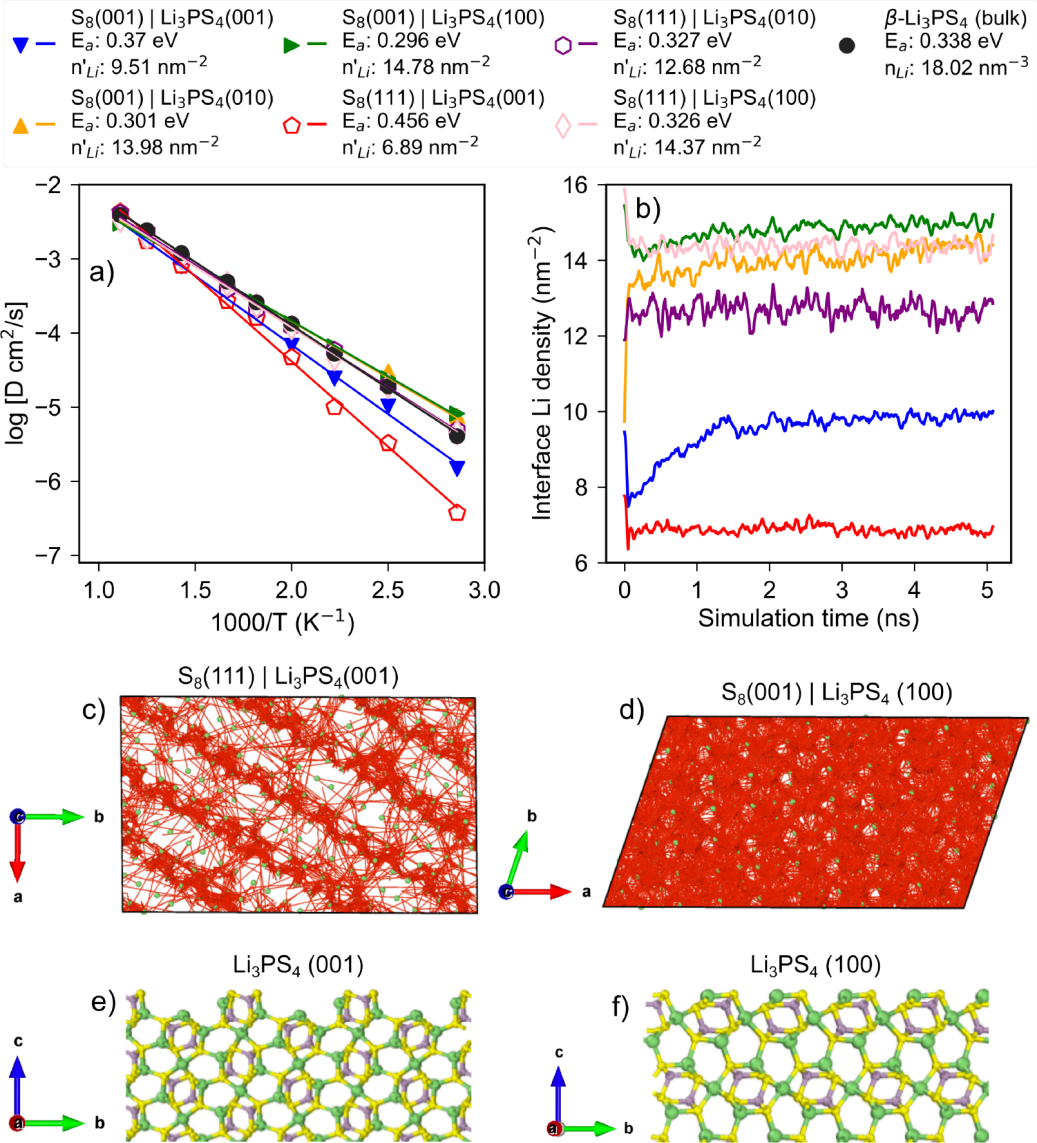
(a) Arrhenius plot and (b) time evolution of Li areal density at the interfacial regions in α-S_8_|β-Li_3_PS_4_ interfaces. The activation barrier for Li-ion migration and average Li areal density *n*_Li_^′^ at the interface region are provided. The Arrhenius plot for bulk β-Li_3_PS_4_ is also provided for reference. Li trajectories (colored in red) from MD simulations for (c) S_8_(111)|Li_3_PS_4_(001) and (d) S_8_(001)|Li_3_PS_4_(100) interfaces viewed along the *c* crystallographic axis (direction perpendicular to interface). Surface morphology of (e) Li_3_PS_4_(001) and (f) Li_3_PS_4_(100) surfaces.

Figure 11a,b show the Arrhenius plots and evolution of the interfacial areal number density of Li *n*_Li_^′^ during simulation time scale for the interface region of the different α-S_8_|β-Li_3_PS_4_ interfaces. For comparison, we have computed the Arrhenius plots of bulk and different surfaces of β-Li_3_PS_4_ in Figure S9a. The *E*_a_ of bulk β-Li_3_PS_4_ is estimated to be 0.338 eV, in agreement with the previously reported value.^82−84^ The (001), (010), and (100) surfaces of β-Li_3_PS_4_ are estimated to have the *E*_a_ values of 0.373, 0.290, and 0.320 eV, respectively. The surface *E*_a_ was obtained from the diffusivity of surface Li atoms of β-Li_3_PS_4_ slabs present in the same interphase thickness range as their derived interfaces. Figure S9b shows the evolution of *n*_Li_^′^ for different β-Li_3_PS_4_ surfaces during the simulation time scale. The β-Li_3_PS_4_ surfaces with lower surface *E*_a_ compared to bulk, i.e., (010) and (100) surfaces, have an estimated *n*_Li_^′^ of ∼13.5 nm^–2^, which is almost twice that of the higher *E*_a_ (001) surface with an *n*_Li_^′^ of 7.5 nm^–2^. The Li diffusion pathways for lower *E*_a_ surfaces are found to be two-dimensional, but those for the higher *E*_a_ (001) surface are predominantly one-dimensional (Figure S9c–e). Hence, it is inferred that surfaces with lower *n*_Li_^′^ and localized Li diffusion pathways tend to have higher surface *E*_a_. In general, we find that the interphase formation results in an increase of *E*_a_ when compared to the parent β-Li_3_PS_4_ surface. However, this is also dependent on the relative Li enrichment/depletion of the interface. For example, S_8_(111)|Li_3_PS_4_(001) has the highest estimated interface *E*_a_ of 0.456 eV, which is ∼0.08 eV higher than the parent β-Li_3_PS_4_(001) surface. Additionally there is a marginal drop in the interface *n*_Li_^′^ value to 6.89 nm^–2^ from 7.5 nm^–2^. Li trajectories of this interface (Figure 11c) also reveal a disruption of the one-dimensional Li diffusion pathways. In contrast, the S_8_(001)|Li_3_PS_4_(001) interface containing the same β-Li_3_PS_4_(001) parent surface shows marginal increase in the *E*_a_ despite the formation of a thicker interphase. It can be observed that the *n*_Li_^′^ for this interface increases over time and averages ∼2 nm^–2^ higher than the parent surface. Similarly, the S_8_(001)|Li_3_PS_4_(100) interface exhibits a marginal drop in the *E*_a_ of ∼0.03 eV compared to the parent β-Li_3_PS_4_(100) surface and has the highest *n*_Li_^′^, which is a marginal increase of ∼1.5 nm^–2^. This is reflected in the marginal increase in the Li trajectories’ coverage when compared to the parent surface (Figure S9e), as seen in Figure 11d.

Finally, it should be noted that while the changes in interface *n*_Li_^′^ and interface Li trajectories can be used to explain the changes in interface *E*_a_, the type of β-Li_3_PS_4_ surface predominantly determines the *E*_a_ at the interface. Surfaces with higher *E*_a_ tend to form interfaces with high *E*_a_. These differences can be traced to the morphology and Li diffusion topology of different β-Li_3_PS_4_ surfaces (Figures 11e,f). The (001) surface is corrugated, which results in the formation of one-dimensional Li diffusion channels with few opportunities for interchannel Li hopping. In contrast, the (100) surface (and (010) surface) is highly planar, resulting in a multitude of diffusion pathways that achieve high coverage of the surface, resulting in lower *E*_a_.

## Conclusion

To conclude, this work provides a comprehensive thermodynamic and kinetic analysis of the cathode/SE interface in LSBs. In general, the charged S_8_ cathode was predicted to be much more reactive than the fully discharged Li_2_S cathode. Among the different anion chemistries, sulfide SEs were found to be the most thermodynamically stable against the LSB cathode. Sulfides are also highly effective buffer layers if the use of other SE anion chemistries is desired for other reasons. We expect these findings to be generalizable to any new SEs or buffer layers discovered for either LIBs or LSBs. Through nanosecond MD simulations using a highly accurate MTP, it was demonstrated that the reactivity at the Li_3_PS_4_/S_8_ interface is related to the relative surface energies of the composite α-S_8_ surfaces; a higher energy surface tends to be more reactivity than a lower energy surface. Finally, while the formation of an interphase tends to increase activation energies for Li diffusion, the surface morphology and Li diffusion topology of the Li_3_PS_4_ surface were found to be the critical determinants of interfacial Li diffusion dimensionality and barriers. The most stable Li_3_PS_4_(100) surface tends to form interfaces with S_8_ with 2D channels and lower activation barriers for Li diffusion.

## Supporting Information Available

Convergence test of MTPs, surfaces of α-S_8_ and β-Li_3_PS_4_, α-S_8_|β-Li_3_PS_4_ interfaces, bond connectivity based clustering illustration, electrochemical reaction energies and corresponding volume change of SE/Li_2_S interfaces, percentage composition vs time for α-S_8_(001) and α-S_8_(111) interfaces, time evolution of spacial distribution of reaction products, activation barrier, time evolution of Li areal number density and surface Li trajectories for β-Li_3_PS_4_ surfaces, source of SE structures, source of oxide and sulfide buffer layers, comparison of experimental, DFT, and MTP cell parameters, training set composition for passive learning, DFT-computed surface energies, interface formation parameters and details, predicted chemical reaction products for SE/cathode interfaces (PDF)
