# Supplementary material for: Thermodynamics and Kinetics of the Cathode–Electrolyte Interface in All-Solid-State Li–S Batteries
Source: J Am Chem Soc. 2022 Sep 23;144(39):18009–22. doi: 10.1021/jacs.2c07482 (PMC9546513; doi:10.1021/jacs.2c07482)
Supplement: Supplementary file 1 — ja2c07482_si_001.pdf [file ja2c07482_si_001.pdf]

Supporting Information: Thermodynamics and Kinetics of the Cathode-Electrolyte Interface in All-Solid-State Li-S Batteries

Manas Likhit Holekevi Chandrappa,[†] Ji Qi,[‡] Chi Chen,[†] Swastika Banerjee,[*,¶] and Shyue Ping Ong[*,†]

†*Department of NanoEngineering, University of California San Diego, 9500 Gilman Dr, Mail Code 0448, La Jolla, CA 92093-0448, United States*

‡*Materials Science and Engineering Program, University of California San Diego, 9500 Gilman Dr, Mail Code 0448, La Jolla, CA 92093-0448, United States*

¶*Department of Chemistry, Indian Institute of Technology Roorkee, Roorkee 247667, India*

E-mail: sbanerjee@cy.iitr.ac.in; ongsp@eng.ucsd.edu

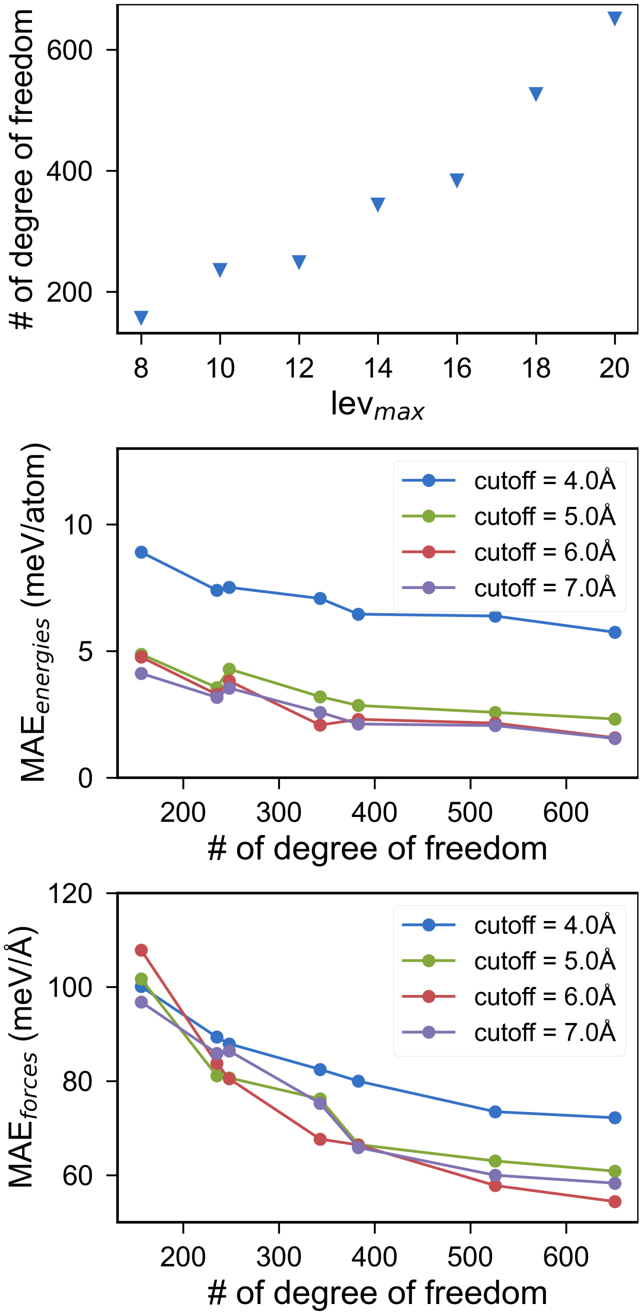

Figure S1: Convergence test of MTP with respect to l_{max} values at different R_{cutoff}. (a) Number of degrees of freedom versus l_{max}. (b) $MAE_{energies}$ and (c) MAE_{forces} versus the number of degrees of freedom.

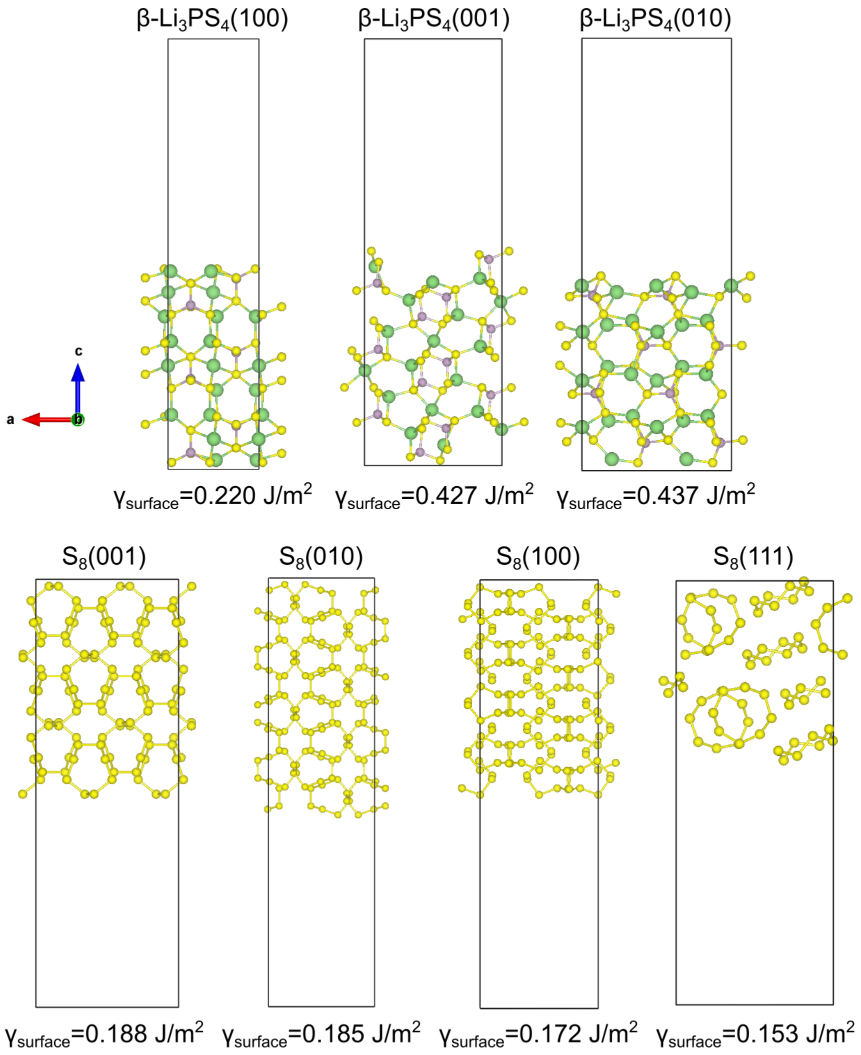

Figure S2: Surface unit cells of β-Li_3PS_4 (top) and α-S_8 (bottom) surfaces with the surface energy values.

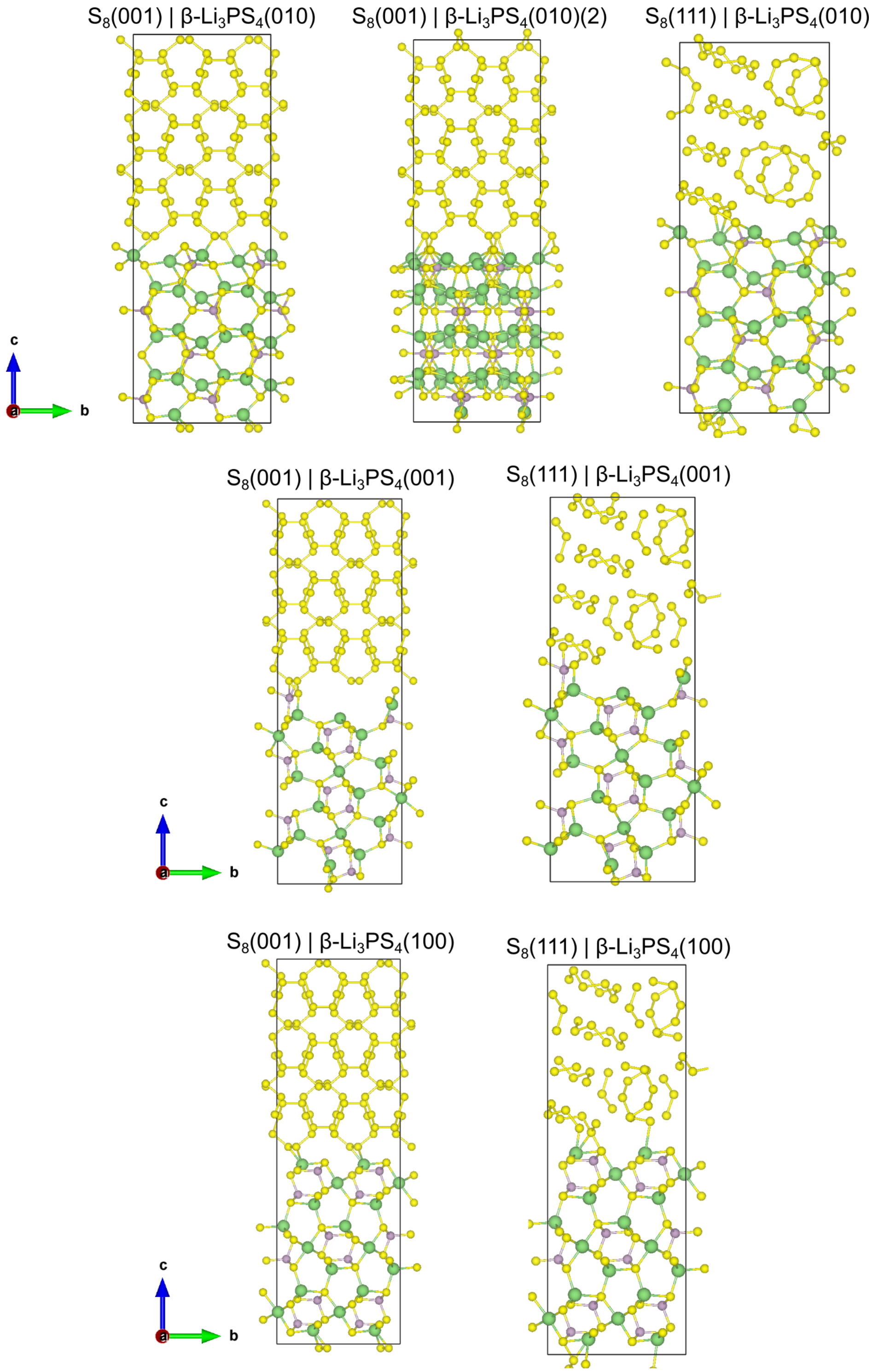

Figure S3: The interface structures constructed by interfacing β-Li_3PS_4(010) (top), β-Li_3PS_4(001) (middle) and β-Li_3PS_4(100) (bottom) surface with α-S_8 surfaces.

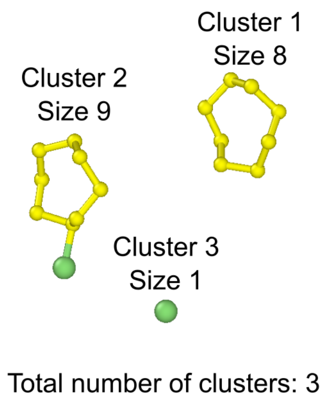

Figure S4: Bond connectivity based atom clustering illustration.

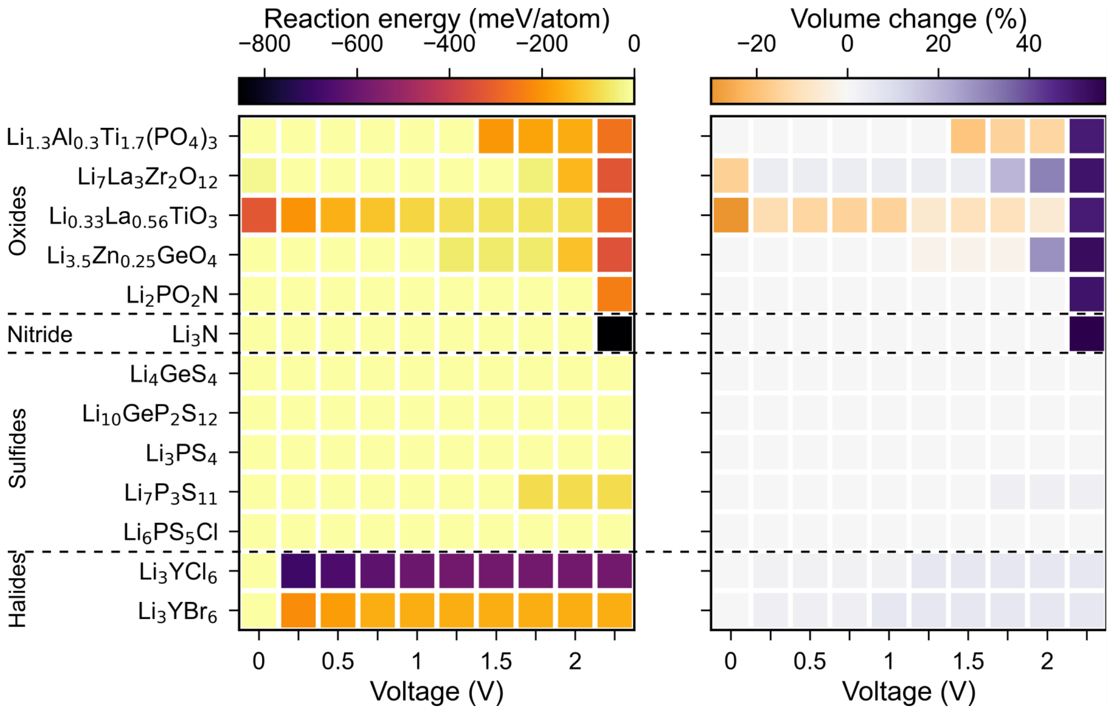

Figure S5: Electrochemical reaction energy (left) and corresponding volume change (right) for Li_2S

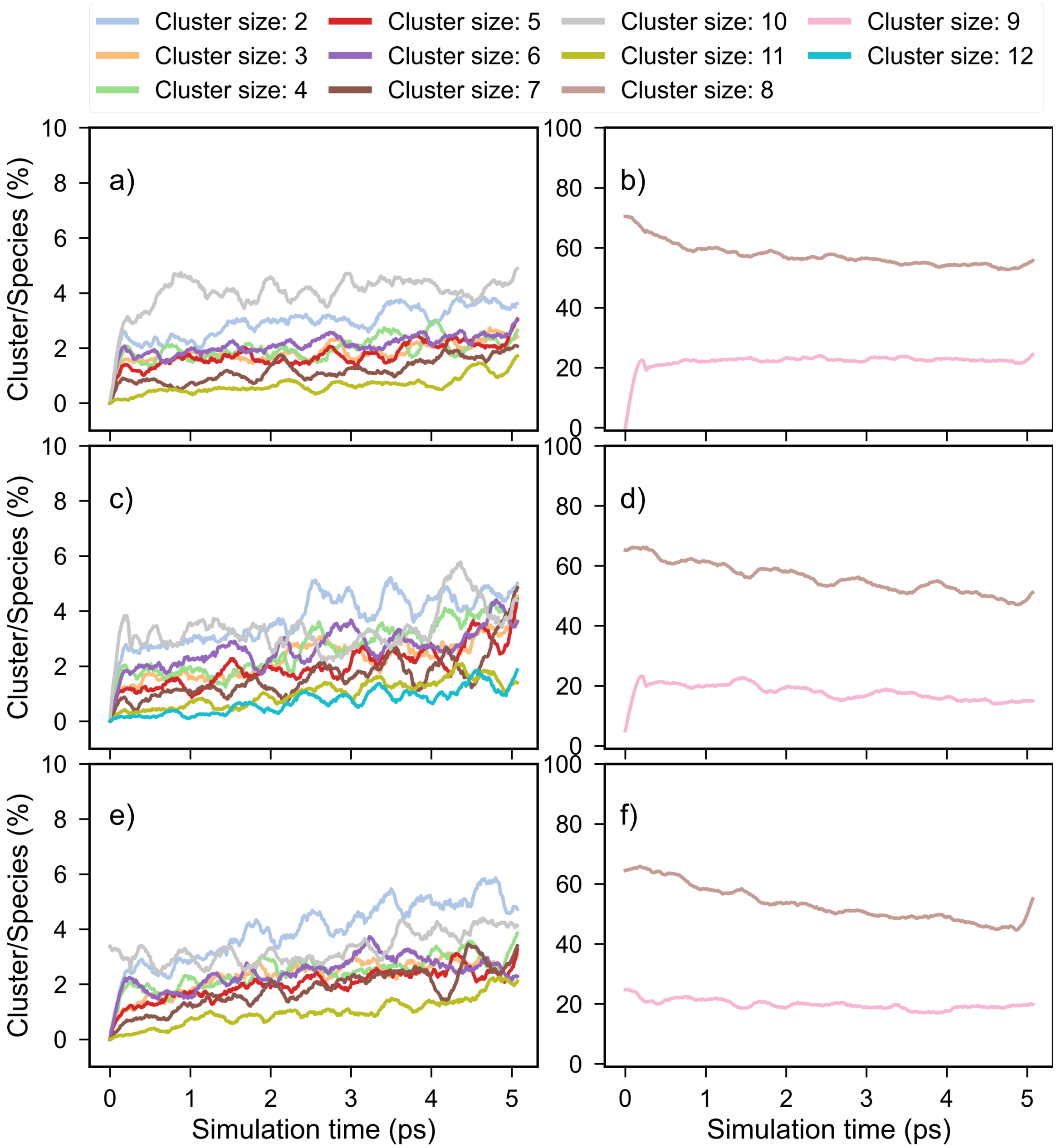

Figure S6: Percentage of cluster sizes at the interface as a function of time for clusters present at <10% (left) and >10% composition in the last 1 ns of the simulation for $S_8(001)|Li_3PS_4(001)$ (top), $S_8(001)|Li_3PS_4(010)$ (middle) and (c) $S_8(001)|Li_3PS_4(100)$ (bottom) interfaces.

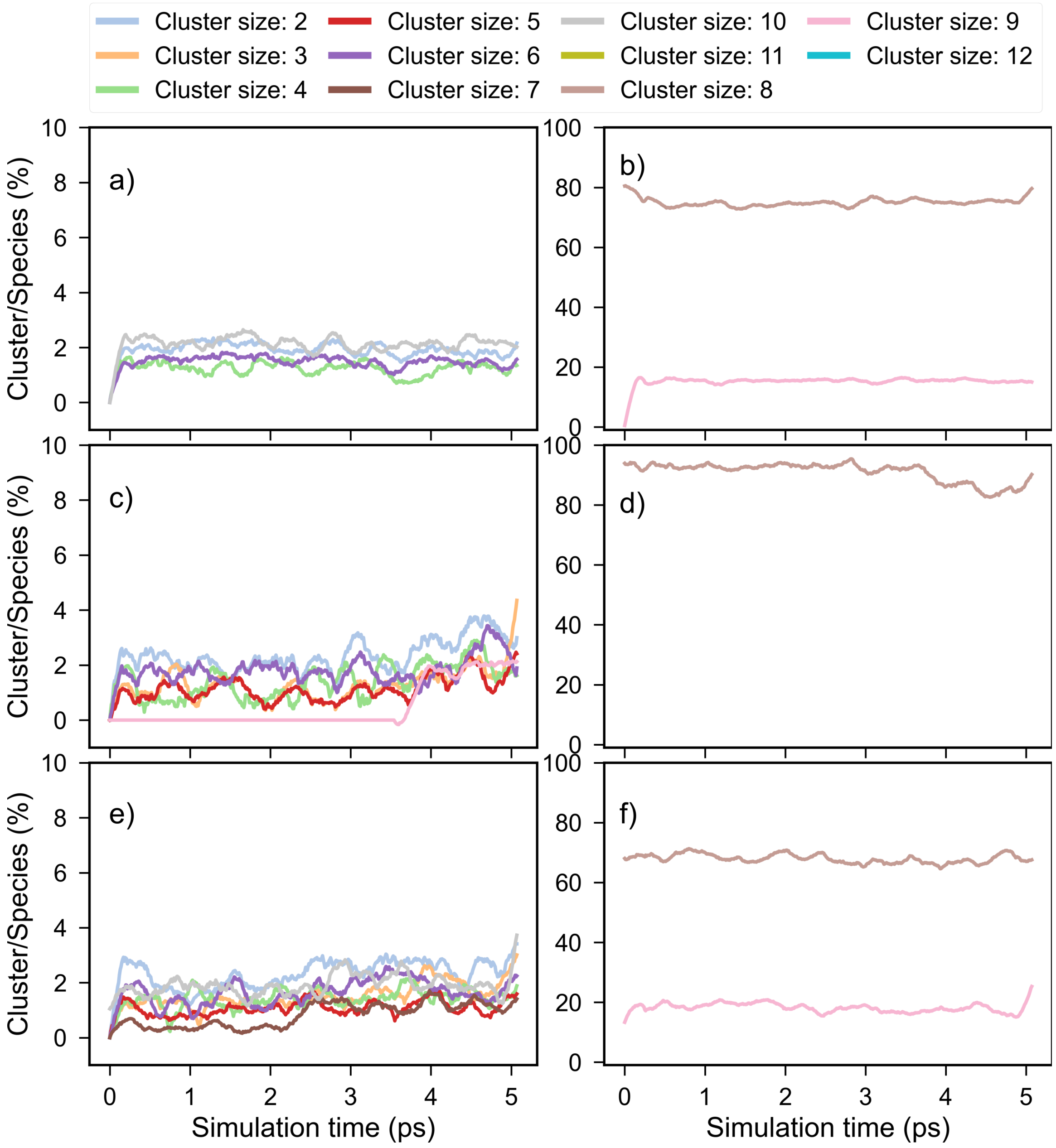

Figure S7: Percentage of cluster sizes at the interface as a function of time for clusters present at <10% (left) and >10% composition in the last 1 ns of the simulation for S_8(111)|Li_3PS_4(001) (top), S_8(111)|Li_3PS_4(010) (middle) and (c) S_8(111)|Li_3PS_4(100) (bottom) interfaces.

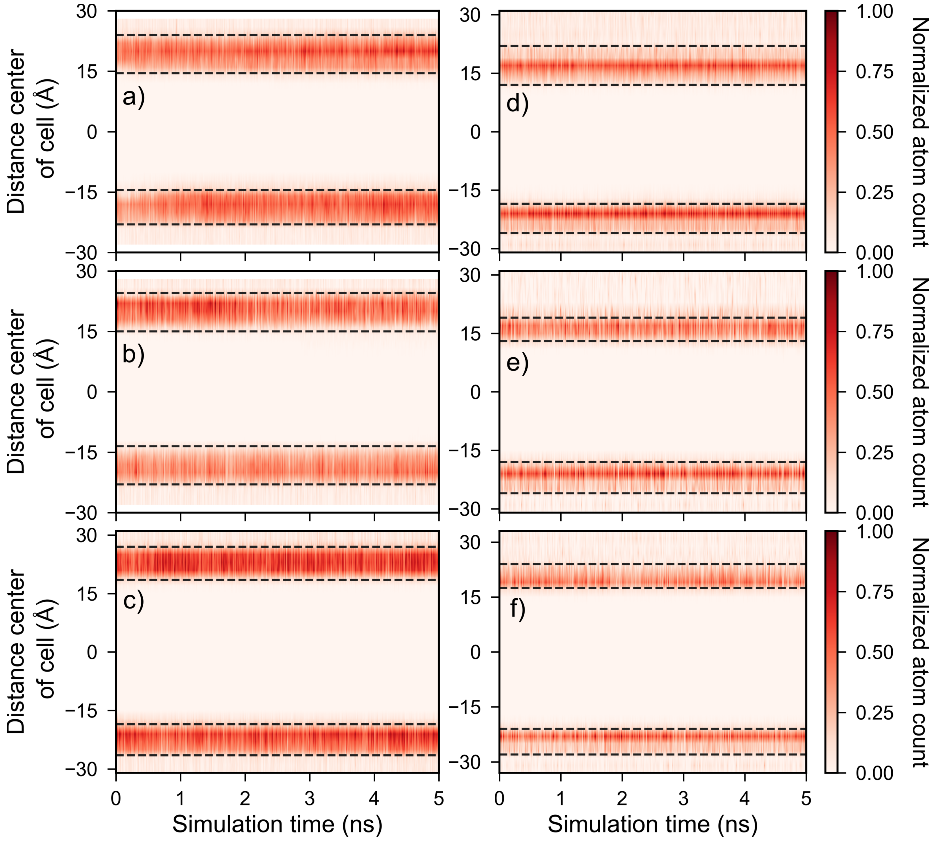

Figure S8: Time evolution of normalized atom count of reactants interface reaction products (cluster size $\neq$ 8) along interface 'c' distance for in (a) S_8(001)|Li_3PS_4(001), (b) S_8(001)|Li_3PS_4(010) and (c) S_8(001)|Li_3PS_4(100) (d) S_8(111)|Li_3PS_4(001), (e) S_8(111)|Li_3PS_4(010) and (f) S_8(111)|Li_3PS_4(100) interfaces.

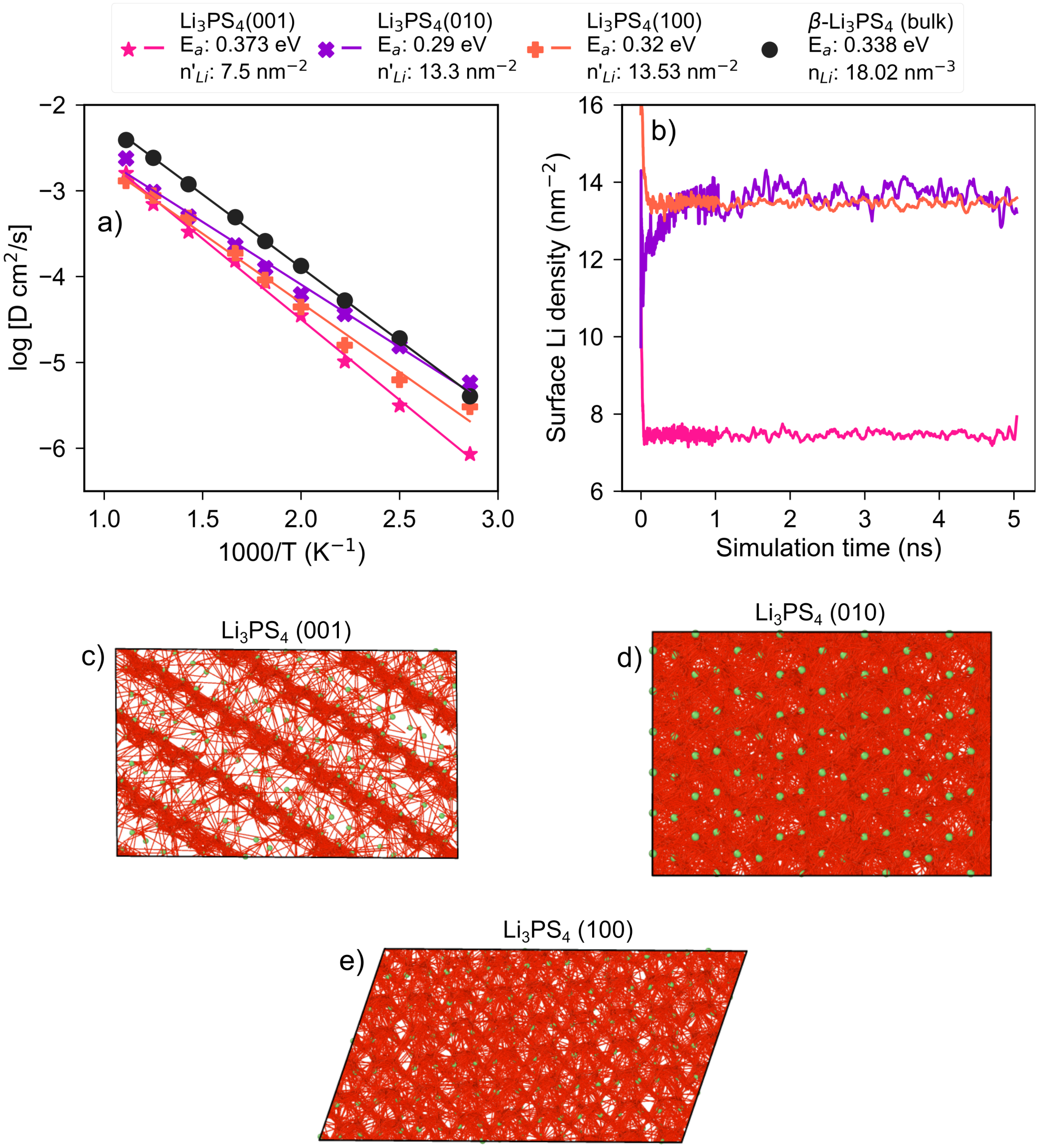

Figure S9: (a) Arrhenius plot (b) time evolution of Li areal density at the β-Li_3PS_4 surfaces. The activation barrier for Li ion migration and average Li areal density at the surface are provided. Arrhenius plot for bulk β-Li_3PS_4 is also provided for reference. Li trajectories (colored in red) from MD simulations for (c) Li_3PS_4(001), (d) Li_3PS_4(010) and (e) Li_3PS_4(100) surfaces.

Table S1: Sources of solid electrolyte structures.

**Solid electrolytes**	**Source**
$Li_{1.3}Al_{0.3}Ti_{1.7}(PO_4)_3$	icsd-427619
$Li_7La_3Zr_2O_{12}$	mp-942733
$Li_{0.33}La_{0.56}TiO_3$	icsd-82670
$Li_{3.5}Zn_{0.25}GeO_4$	icsd-100169
Li_2PO_2N	icsd-188493
Li_3N	mp-2251
Li_4GeS_4	mp-30249
$Li_{10}GeP_2S_{12}$	mp-696138
Li_3PS_4	icsd-180319
$Li_7P_3S_{11}$	mp-641703
Li_6PS_5Cl	icsd-259200
Li_3YCl_6	icsd-259202
Li_3YBr_6	icsd-259212

Table S2: Sources of Oxide buffer layers along with the computed e_above_hull values

**Oxide buffer layers**	**Source**	**e_above_hull(eV)**
$Li_4Ti_5O_{12}$	mp-685194	0.0
$LiNbO_3$	mp-3731	0.0
$LiTaO_3$	mp-3666	0.0
$Li_2Ti_2O_5$	mp-772049	0.03
$Li_4Ti_5O_{12}$	mp-685194	0.0
Li_2ZrO_3	mp-4156	0.0
LiH_2PO_4	mp-24610	0.0

Continued on next page

Table S2 continued from previous page

**Oxide buffer layers**	**Source**	**e_above_hull(eV)**
$LiTi_2(PO_4)_3$	mp-25840	0.0
$LiBa(B_3O_5)_3$	mp-558890	0.0
$LiPO_3$	mp-1202801	0.0
$LiLa(PO_3)_4$	mp-560866	0.0
$LiCs(PO_3)_2$	mp-560667	0.0
Li_2SiO_3	mp-5012	0.0
Li_3PO_4	mp-13725	0.0
Li_4TiO_4	mp-9172	0.0
Li_2TiO_3	mp-2931	0.0
$Li_8Nb_2O_9$	mp-28030	0.0
Li_3NbO_4	mp-31488	0.0
$LiNb_3O_8$	mp-3368	0.0
Li_8SiO_6	mp-28549	0.0
Li_4SiO_4	mp-1223129	0.0
$Li_2Si_2O_5$	mp-4117	0.0
Li_5TaO_5	mp-755013	0.0
Li_3TaO_4	mp-3151	0.0
$LiTa_3O_8$	mp-7638	0.0

Continued on next page

Table S2 continued from previous page

**Oxide buffer layers**	**Source**	**e_above_hull(eV)**
$Li_4P_2O_7$	mp-28450	0.0
$LiAlO_2$	mp-3427	0.0
Li_3BO_3	mp-27275	0.0
Al_2O_3	mp-1143	0.0
ZnO	mp-2133	0.0
CdO	mp-1132	0.0
Sc_2O_3	mp-216	0.0
Y_2O_3	mp-2652	0.0
La_2O_3	mp-2292	0.0
SiO_2	mp-546794	0.0
TiO_2	mp-554278	0.0
ZrO_2	mp-2858	0.0
HfO_2	mp-352	0.0
Nb_2O_5	mp-581967	0.0
Ta_2O_5	mp-1539317	0.0

Table S3: Sources of Sulfide buffer layers along with the computed e_above_hull values

**Sulfide buffer layers**	**Source**	**e_above_hull(eV)**
Li_2SiS_3	mp-1210789	0.0
Li_3PS_4	mp-985583	0.0
Li_4TiS_4	mp-766540	0.0
Li_3NbS_4	mp-769032	0.0
$LiAlS_2$	mp-1106183	0.0
Li_3BS_3	mp-5614	0.0
Al_2S_3	mp-2654	0.0
ZnS	mp-556716	0.0
CdS	mp-672	0.0
Sc_2S_3	mp-401	0.0
Y_2S_3	mp-541289	0.0
La_2S_3	mp-7475	0.0
SiS_2	mp-1602	0.0
TiS_2	mp-2156	0.0
ZrS_2	mp-1186	0.0
HfS_2	mp-985829	0.0

Table S4: Comparison of PBE and optB88 computed lattice parameters with experimental values for β-Li_3PS_4, α-S_8 and Li_2S. The percentage error with respect to experimental value is provided in the parenthesis.

**Cell parameters**	**Experimental**	**PBE**	**optB88**
		β-Li_3PS_4	
a (Å)	12.819	13.083 (2.06)	13.051 (1.81)
b (Å)	8.219	8.130 (-1.08)	7.951 (-3.26)
c (Å)	6.123	6.270 (2.39)	6.149 (0.43)
		α-S_8	
a (Å)	10.464	11.889 (13.61)	10.333 (-1.25)
b (Å)	12.866	14.160 (10.06)	12.829 (-0.28)
c (Å)	24.485	26.076 (6.49)	24.502 (0.06)
		Li_2S	
a (Å)	5.720	5.721 (0.02)	5.689 (-0.54)
b (Å)	5.720	5.721 (0.02)	5.689 (-0.54)
c (Å)	5.720	5.721 (0.02)	5.689 (-0.54)

Table S5: The composition of the training set used for passive learning.

**Compound**	**Type**	**Temperature**	**Number of structures**
Li_2S	bulk	300, 600, 900, 1200	4 temps × 10 = 40
	strained bulk	300, 600, 900, 1200	4 temps × 10 = 40
	slab (111), (011), (110)	-	3 surfaces × 10 = 30
	stained slab (111), (011), (110)	-	3 surfaces × 10 = 30
S_8	bulk	300, 600, 900, 1200	4 temps × 10 = 40
	strained bulk	300, 600, 900, 1200	4 temps × 10 = 40
	slab (001), (010), (100),(111)	-	4 surfaces × 10 = 40
	stained slab (001), (010), (100),(111)	-	4 surfaces × 10 = 40
Li_3PS_4	bulk	300, 600, 900, 1200	4 temps × 10 = 40
	strained bulk	300, 600, 900, 1200	4 temps × 10 = 40
	melted	2000	1 temps × 10 = 10
	strained melted	2000	1 temps × 10 = 10
	slab (001), (100)	-	2 surfaces × 2 = 4
	stained slab (001), (100)	-	2 surfaces × 10 = 20
Total number of structures			384

Table S6: DFT computed surface energies of different of β-Li_3PS_4 and α-S_8 surfaces.

**Surface**	**Surface energy (J/m^2)**
β-Li_3PS_4	
100	0.371
001	0.608
010	0.620
α-S_8	
001	0.188
010	0.185
100	0.172
111	0.153

Table S7: Details of interfaces formed using different surfaces of β-Li_3PS_4 and α-S_8 along with DFT interfacial energies.

**β-Li_3PS_4 surface**	**α-S_8 surface**	**Mismatch (%)**	**relative rotation**	**No. of atoms**	**Slab distance**	**Interface energy (J/m^2)**
100	001	6.4	0.0	192	1.6	33.16
	111	5.8	0.0	160	1.6	22.88
001	001	6.4	0.0	192	1.0	34.15
	111	5.9	0.0	160	0.1	41.89
010	001	7.8	0.0	160	1.0	39.12
	001	10.0	90.0	160	1.7	40.82
	111	7.8	0.0	256	0.7	57.58

Table S8: Predicted reaction products at SE interfaces with S_8 and Li_2S.

SE	S_8 reaction products	Li_2S reaction products
$Li_{1.3}Al_{0.3}Ti_{1.7}(PO_4)_3$	TiS_3, S_8O, P_2S_7, Li_3PS_4, $LiAlS_2$	TiS_2, Li_3PS_4, Li_3PO_4, $LiAl_5O_8$
$Li_7La_3Zr_2O_{12}$	LaS_2, ZrS_3, Li_2S, S_8O	-
$Li_{0.33}La_{0.56}TiO_3$	LaS_2, TiS_3, S_8O, Li_2S	Li_2TiO_3, $La_{10}S_{19}$, $Li(TiS_2)_2$, $La_{10}S_{14}O$
$Li_{3.5}Zn_{0.25}GeO_4$	Li_2ZnGeS_4, Li_4GeS_4, S_8O	Li_4GeO_4, ZnS
Li_2PO_2N	Li_3PS_4, S_7N, P_2S_7, S_8O	-
Li_3N	N_2, Li_2S	-
Li_4GeS_4	-	-
$Li_{10}GeP_2S_{12}$	-	-
Li_3PS_4	-	-
$Li_7P_3S_{11}$	Li_3PS_4, P_2S_7	Li_3PS_4
Li_6PS_5Cl	-	-
Li_3YCl_6	-	$LiYS_2$, LiCl
Li_3YBr_6	Y2S3, LiBr, SBr	$LiYS_2$, LiBr

Table S9: Comparison of 0K DFT and MTP computed cell parameters and energies for polymorphs of Li_3PS_4. MTP errors with respect to the DFT values are provided in parentheses.

**Phase**	**Quantity**	**DFT**	**MTP**
α	a (Å)	8.546	8.489 (0.015)
	b (Å)	9.317	9.255 (0.139)
	c (Å)	8.345	8.288 (0.038)
	Energy (eV/atom)	-4.200	-4.201 (-0.001)
	$E_{phase-\beta}$ (eV/atom)	-0.012	-0.014 (-0.002)
β	a (Å)	13.083	12.995 (-0.056)
	b (Å)	8.13	8.082 (0.131)
	c (Å)	6.27	6.230 (0.081)
	Energy (eV/atom)	-4.212	-4.216 (-0.004)
γ	a (Å)	7.778	7.779 (0.081)
	b (Å)	6.625	6.626 (0.061)
	c (Å)	6.235	6.236 (0.054)
	Energy (eV/atom)	-4.227	-4.227 (-0.000)
	$E_{phase-\beta}$ (eV/atom)	0.014	0.011 (-0.003)
